# Overlap and Specificity in the Substrate Spectra of Human Monoamine Transporters and Organic Cation Transporters 1, 2, and 3

**DOI:** 10.3390/ijms222312816

**Published:** 2021-11-26

**Authors:** Lukas Gebauer, Ole Jensen, Maria Neif, Jürgen Brockmöller, Christof Dücker

**Affiliations:** Institute of Clinical Pharmacology, University Medical Center Göttingen, D-37075 Göttingen, Germany; ole.jensen@med.uni-goettingen.de (O.J.); maria.neif@stud.uni-goettingen.de (M.N.); jbrockm@gwdg.de (J.B.); christof.duecker@med.uni-goettingen.de (C.D.)

**Keywords:** SLC6, monoamine transporters, organic cation transporters, biogenic amines, matched molecular pair analysis, substrates

## Abstract

Human monoamine transporters (MATs) are cation transporters critically involved in neuronal signal transmission. While inhibitors of MATs have been intensively studied, their substrate spectra have received far less attention. Polyspecific organic cation transporters (OCTs), predominantly known for their role in hepatic and renal drug elimination, are also expressed in the central nervous system and might modulate monoaminergic signaling. Using HEK293 cells overexpressing MATs or OCTs, we compared uptake of 48 compounds, mainly phenethylamine and tryptamine derivatives including matched molecular pairs, across noradrenaline, dopamine and serotonin transporters and OCTs (1, 2, and 3). Generally, MATs showed surprisingly high transport activities for numerous analogs of neurotransmitters, but their substrate spectra were limited by molar mass. Human OCT2 showed the broadest substrate spectrum, and also the highest overlap with MATs substrates. Comparative kinetic analyses revealed that the radiotracer meta-iodobenzylguanidine had the most balanced uptake across all six transporters. Matched molecular pair analyses comparing MAT and OCT uptake using the same methodology could provide a better understanding of structural determinants for high cell uptake by MATs or OCTs. The data may result in a better understanding of pharmacokinetics and toxicokinetics of small molecular organic cations and, possibly, in the development of more specific radiotracers for MATs.

## 1. Introduction

The monoamine neurotransmitters dopamine, norepinephrine, and serotonin play essential roles in motor control, cognition, memory processing, emotion, and many other body functions [1]. Monoamines act on extracellular binding sites of several receptors [2] and signaling is terminated by transporter-mediated uptake with subsequent intracellular storage and recycling or enzyme-mediated degradation. Two systems mainly acting as monoamine reuptake systems have been identified and historically classified as uptake1 and uptake2 [3,4].

Uptake1 is formed by the human monoamine transporters (MATs), including the norepinephrine (NET/SLC6A2), dopamine (DAT/SLC6A3), and serotonin transporter (SERT/SLC6A4). MATs are crucial in terminating the transmission of monoaminergic neurons [5]. They are mainly, but not exclusively, expressed at distinct regions in the brain and are characterized by a high affinity to their name-giving substrates. NET is an exception since it shows an even higher affinity towards dopamine than towards the name-giving norepinephrine itself [6]. All MATs are promiscuous and capable of transporting the other monoamine neurotransmitters as well [7]. Monoamine reuptake inhibitors with differential selectivity have been developed and are established treatment options in conditions such as depression, anxiety disorder, obsessive-compulsive disorder, and attention deficit hyperactivity disorder [8]. Besides approved drugs, many recreationally used psychostimulants exert their function at monoamine transporters. Pharmacodynamically speaking, those psychostimulants are either predominantly reuptake inhibitors (e.g., cocaine) or substrate-type releasers (e.g., amphetamine) [9].

The second monoamine reuptake system, uptake2, has later been identified to include the organic cation transporters 1, 2, and 3 (OCTs) of the SLC22 family. OCTs transport a broad spectrum of hydrophilic organic cations [10], including, but not limited to, monoamine neurotransmitters. Apart from transporting endogenous substrates, OCTs play a major role in transporting exogenous compounds including numerous approved drugs [11,12,13,14,15,16]. Initially, uptake2 had been characterized as a low-affinity but high-capacity transport system in peripheral tissues [17]. Meanwhile, it has been established, that OCT2 and OCT3 are also expressed in the central nervous system and under certain circumstances modulate monoaminergic signaling [18,19,20,21,22]. In addition, as will be shown in this study, for several substrates the contrary is true, namely, MATs may have a substantially higher capacity than the OCTs. 

While inhibitors of MATs—as well as OCTs—have been studied intensively [23,24,25,26,27] and ligand- as well as structure-based determinants of inhibition have been identified [28,29,30,31], less is known about the substrate spectra of MATs and OCTs. Especially with MATs, relatively little data is available beyond the narrow substrate group of the neurotransmitters themselves. From the limited number of earlier identified substrates of MATs, several have been identified only based on indirect indicators of possible transport such as transporter inhibition assays (in some cases combined with assays on substrate release) [26,32,33,34] or by electrophysiological methods [35,36]. Only very few studies have characterized the substrate spectrum of MATs using direct measurements by radiolabeled compounds or LC/MS-MS analyses of cell uptake of the substrates [37].

Given that MATs and OCTs, uptake1 and uptake2, have been discovered as two players in the same process, astonishingly little effort has been made to compare them directly, regarding structure-activity relationships. From a basic research perspective, a more systematic evaluation of the substrate overlap of both transporter families could lead to a better understanding of monoamine (brain) physiology as well as a better understanding of the fundamental biological roles of the OCTs. Additionally, and from a more applied clinical perspective, the identification of the substrate overlap between MATs and OCTs is of great interest for the development of selective radiotracers for diagnostics and therapy [38]. Meta-iodobenzylguanidine (mIBG), a NET tracer used for diagnostics in heart diseases [39] as well as for diagnostics and therapy in neuroendocrine cancer patients [40], has relatively recently been proven to be an excellent OCT substrate [41,42]. High affinity towards OCTs explains some difficulties in mIBG diagnostics and treatment that were previously not understood on a molecular level.

Recently, we investigated the uptake of different psychostimulants, showing that there is overlap between MATs and OCTs in the uptake of substances which in their chemical structure differ from the typical neurotransmitters. Particularly cathine was transported by both the MATs (DAT and NET), but also by OCT2 [43]. As opposed to OCT2, OCT1 transported only one of the tested psychostimulants to a significant degree, namely mescaline. This may indicate that with all known substrate polyspecificity encountered in OCTs, there may exist substances that are exclusively taken up into the cell by MATs. In addition, as it will be shown in this study, it may well be that OCT2 has the broadest substrate spectrum among the OCTs with respect to small organic cations. With the psychostimulants investigated, no conclusive molecular descriptors differentiating between MAT and OCT substrates could be derived, as these psychostimulants were molecularly too heterogeneous to allow for meaningful pairwise comparisons [43]. Therefore, we here wanted to derive these predictors by comparative uptake analyses of numerous substances with a phenylethylamine- or tryptamine-based backbone structurally closely related to the monoamine neurotransmitters. For this, we comparatively screened the candidate compounds for uptake by MATs and OCTs. We then analyzed whether general chemical descriptors are sufficient to explain differences in the uptake patterns of both transporter families. Finally, we carried out a series of matched molecular pair analyses to point out which molecular features are required for efficient uptake by the distinct transporters.

## 2. Results

**Figure 1:**
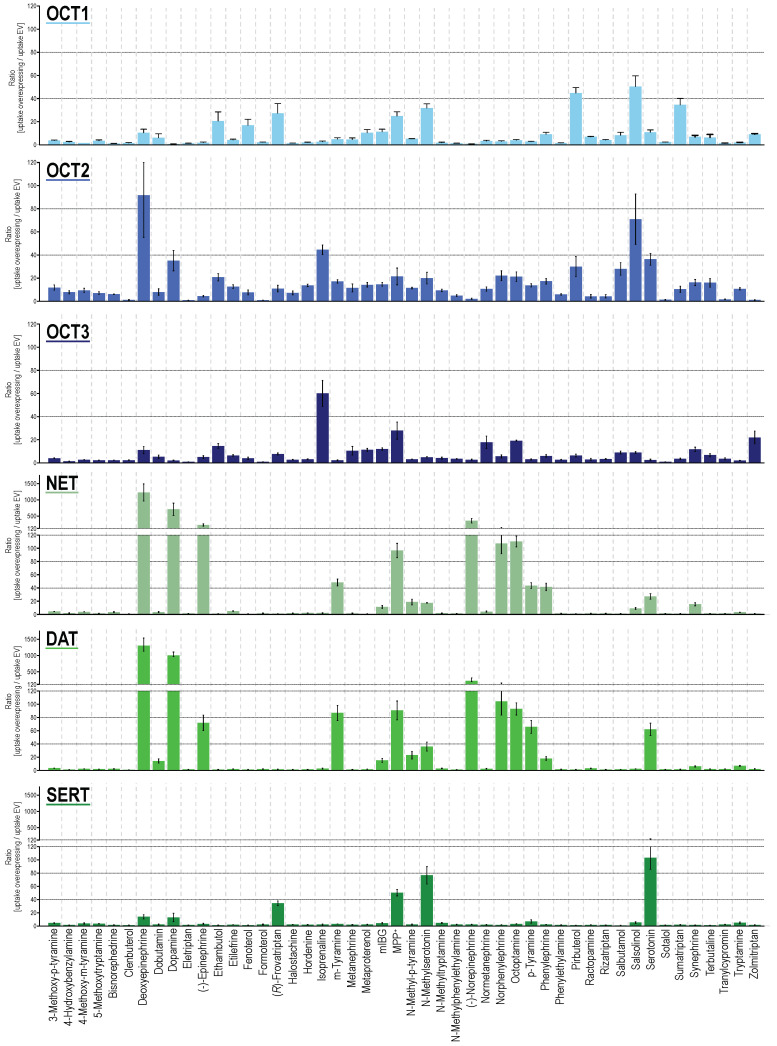
Transport ratios, uptake into overexpressing cells over uptake into empty-vector control cells for compounds as shown on the x-axis (alphabetical order). HEK293 cells were incubated with 2.5 μM of each respective compound for two minutes (five minutes for epinephrine and norepinephrine). Data are provided as the means with error bars indicating the SEM of at least three independent experiments. Uptake ratios were tested for statistical significance using one sample *t*-tests with an α-value of 0.05 as reported in Table S1.

From the 48 substances tested, 33, 32, 35, 28, 26, and 18 were taken up significantly by OCT1, OCT2, OCT3, NET, DAT and SERT, when comparing cell uptake in overexpressing cells with mock-transfected cells (Figure 1 and Figure S1, Table S1). When using an uptake ratio of 3 as cutoff, the corresponding numbers were 29, 41, 32, 23, 18, and 17 and thus, apparently, OCT2 had by far the broadest substrate spectrum. At least for OCT1, the cutoff of 3 has previously been shown to successfully discriminate compounds as substrates and non-substrates in accordance with transport kinetic parameters [42]. Overall, the OCTs had a broader substrate spectrum compared with MATs. Nevertheless, MATs had a surprisingly strong uptake capacity for several substances beyond their “classical” neurotransmitter substrates. For several substrates, MATs are clearly not low-capacity transporters, but of course, in vivo, relative tissue expressions have to be taken into account.

As expected from sequence homologies, NET and DAT showed the most similar uptake pattern among the six investigated transporters. Both showed transport ratios even upwards from 100, for their best substrates, which, apart from their eponymous neurotransmitters, also included deoxyepinephrine and norphenylephrine (Figure 1). With slightly lower transport ratios, the same could be observed for SERT. With SERT, besides serotonin, *N*-methyl serotonin showed an uptake ratio around 100. For OCTs, maximum transport ratios were lower, with most of the best substrates reaching uptake ratios of around 50, notable exceptions being deoxyepinephrine with an uptake ratio of 92 (OCT2) and salsolinol with an uptake ratio of 71 (OCT2). Apart from this, serotonin (OCT2) and neurotransmitter-similar compounds such as isoprenaline (for OCT2 and 3), deoxyepinephrine as well as dopamine (OCT2), and *N*-methyl serotonin (OCT1), showed the highest transport ratios. Those substances with phenethylamine or tryptamine-based backbone but larger substitutions, clinically used as monoamine agonists (α- and β-adrenergic, as well as serotonergic agonists), were efficiently transported by OCTs although in a different pattern among the three transporters. With respect to specificity, sumatriptan and pirbuterol showed the highest transporter-specific uptake by OCT1. For OCT2 and OCT3, salbutamol as well as hordenine, and zolmitriptan as well as isoprenaline were the most specific substrates, respectively (Figure S2A). For the MATs, epinephrine, dopamine, and serotonin were the most specific substrates for NET, DAT, and SERT as expected given their physiological functions. A global comparison of the generated uptake dataset revealed that, besides NET and DAT ratios (r = 0.978), also the uptake ratios for OCT2 correlated well with those of NET and DAT (r = 0.615 and 0.625) even better as with those for OCT1 (r = 0.428, Figure S2B). SERT uptake ratios showed highest correlation with OCT1 uptake (r = 0.306) and OCT3 uptake correlated best with OCT2 values (r = 0.322).

Larger compounds mainly showed little to no transport via MATs. Interestingly, there was one exception for this, namely frovatriptan, with an uptake ratio of 34 despite a larger compound size. Frovatriptan, a serotonin receptor agonist, with a molar mass of 243 Da (as compared to serotonin with a molar mass of 176 Da), does not only differ from serotonin by a hydroxy-to-amide substitution, but also by a ring closure (Figure S3). 

As frovatriptan appeared to be an unusual SERT substrate, concentration-dependent experiments were performed. Comparative uptake of frovatriptan and serotonin into SERT-overexpressing cells showed similar transport capacity for both but an increased K_m_ with frovatriptan (Figure S3). Generally, high uptake rates of serotonin and N-methyl serotonin were found for all transporters except OCT3 and therefore these two compounds were analyzed more thoroughly by concentration-dependent transport experiments (Figure S4A). Results showed saturable uptake by OCT1 and 2 and all three MATs, but not by OCT3. The kinetic analysis confirmed that serotonin N-methylation greatly improved its OCT1 transport and also uptake via OCT3 (Figure S4A). OCT2 transport capacity is reduced by N-methylation and DAT and SERT transport are almost unaffected, while NET transport is characterized by a reduced transport capacity (Figure S4B). It is to note that intrinsic clearances by OCT1 for both substrates were well within the range of NET and DAT, and only topped out by SERT. Moreover, a remarkably high capacity of OCT2 uptake for serotonin as well as for N-methyl serotonin was found, with vmax values of 31,565 pmol × mg protein^−1^ × min^−1^ and 11,918 pmol × mg protein^−1^ × min^−1^, respectively (Table S2).

To analyze whether basic chemical descriptors are sufficient to explain the observed differences in the uptake by both transporter families, we compared those descriptors (for compounds transported with transport ratios of ≥3) across all MATs and OCTs. Main differences were observed for several descriptors related to molecular size, including molar mass, heavy atom molar mass, and heavy atom count (Table S3). That makes interpretation of other differences, such as in the number of rotatable bonds hard to interpret, as those are overall also correlated with molar mass. Albeit to some extent based on our specific compound selection, the number of compounds with transport ratios ≥3 was the highest for OCT2. 

**Figure 2:**
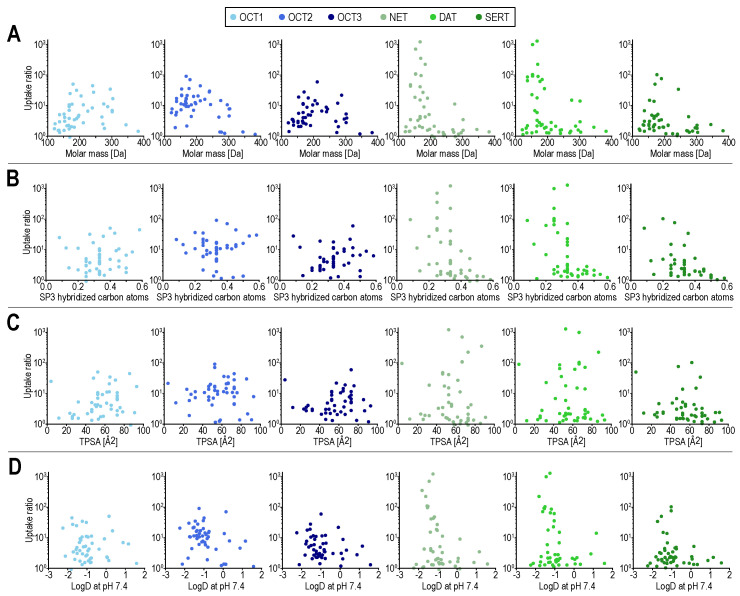
Transport ratios (uptake into overexpressing cells over uptake into empty-vector control cells) for all compounds on the ordinate. On the abscissae molar mass (**A**), fraction of SP3 hybridized atoms (**B**), total polar surface area (TPSA) in (**C**), and logD (at pH 7.4) in (**D**). Scatter colors indicate the respective transporters, with MATs in green and OCTs in blue. In (**B**) ethambutol, as an outlier, with a fraction of SP3 hybridized carbon atoms of 1 is not shown. LogD at pH 7.4 was calculated using the software cxcalc from ChemAxon, Budapest, Hungary, the remaining molecular descriptors were calculated using RDKit, RDKit: open-source cheminformatics software, https://www.rdkit.org/ (accessed on 7 October 2021).

Compared with OCTs, the MAT transport seems to be more limited by compound size. Chemical descriptors such as molar mass and fraction of SP3 hybridized atoms are apparently not sufficient to define the substrate spectrum of MATs and to differentiate it from the substrate spectrum of OCTs (Figure 2). While it appears to be relatively easy to find an OCT substrate that is not a MAT substrate, e.g., by searching for positively charged substances with a molar mass larger than 250 Da that are OCT substrates, finding MAT substrates that are not OCT substrates, cannot be solved this way. This is because, on the one hand, small compounds are not necessarily good MAT substrates and on the other hand, they are not necessarily bad OCT substrates.

**Figure 3:**
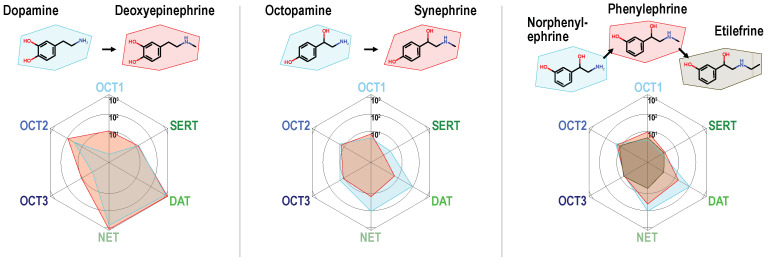
Alkylation of the primary amino group in phenylethylamines reduced uptake ratios of monoamine transporters and increased uptake by OCT1. Single *N*-methylated compounds are still good MAT substrates, but larger substituents as shown for the ethyl chain in etilefrine strongly reduce MAT transport.

The basic chemical descriptors were not able to discriminate between compounds showing low or high uptake by MATs. Therefore, we carried out-matched molecular pair analyses (complete overview in Figure S5), to find further molecular determinants of MAT-specific uptake. Compounds were aligned in pairs that differ only by a single molecular change. Based on these comparisons, the following structure–activity relationships were observed: For the phenylethylamine derivatives, branched or non-branched *N*-methylation generally reduced uptake by MATs (albeit from a normally high level of transport). Interestingly, as can be seen using the direct comparisons, the same substitutions increased uptake by OCT1 and in some cases by OCT3 (Figure 3). It is worth noting that for uptake by MATs, the single methylation is still tolerated, whereas, for instance, the slightly larger ethyl chain of etilefrine results in very low MAT uptake ratios.

**Figure 4:**
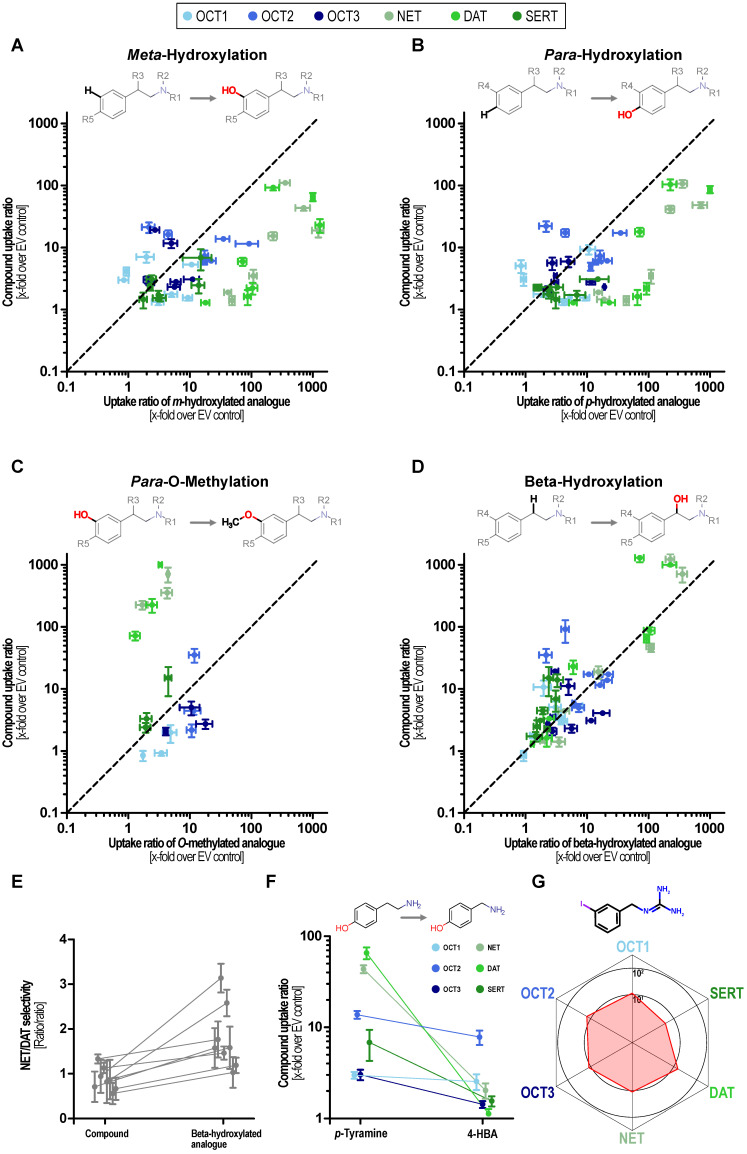
Structure–activity relationship analyses of phenylethylamine-based substrates. Effects of *meta*- (**A**) and *para*-hydroxylation (**B**), methylation of *para*-hydroxy groups (**C**), and beta-hydroxylations (**D**) on transport ratios for MATs and OCTs. The uptake ratio of the parent compound is shown on the ordinate and the uptake ratio of the corresponding substituted compound is displayed on the x-axis. Pairs above the bisection indicate that the uptake ratio is reduced by the respective substitution and pairs below the bisection indicate that the uptake ratio increased it. (**E**) Influence of beta-hydroxylation on NET/DAT selectivity. (**F**) Comparison of uptake ratios of *p*-tyramine and 4-hydroxybenzylamine (4HBA). (**G**) Balanced uptake ratio of the NET-tracer mIBG by MATs and OCTs.

Dopamine serves as an exception of this observation as the *N*-methylated analog deoxyepinephrine shows similarly high MAT uptake-ratios. Hydroxylations at the phenyl ring at either the *meta* or the *para* site or at both, greatly improved transport via MATs, which has been known but now can be compared with its effect on OCTs. These did not improve transport at OCTs to a similar extent as they did in MATs and thereby improved the relative specificity towards MATs (Figure 4A,B).

MAT transport was almost completely abolished by methylation of the catechol hydroxy groups (Figure 4C). Beta-hydroxylation slightly increased transport ratios by NET except for dopamine and *N*-methyl dopamine. DAT ratios were unaffected or even reduced by beta-hydroxylation thereby shifting the NET/DAT selectivity towards NET (Figure 4D,E). SERT uptake ratios were reduced by beta-hydroxylation and OCT ratios were not affected in a systematic manner. As a note, the direct comparison of transport ratios for *p*-tyramine and 4-hydroxybenzylamine suggests that the distances of the aromatic ring and the positively charged nitrogen are crucial for MAT transport (Figure 4F). Interestingly, the NET tracer *meta*-iodbenzylguanidin (mIBG) showed the most homogenous uptake with a ratio of 10 for the OCTs and NET as well as DAT while the SERT uptake ratio was slightly lower (Figure 4G). Given this finding and the prominent role of mIBG as a clinically applied NET tracer, we carried out concentration-dependent uptake analyses by all six transporters.

**Figure 5:**
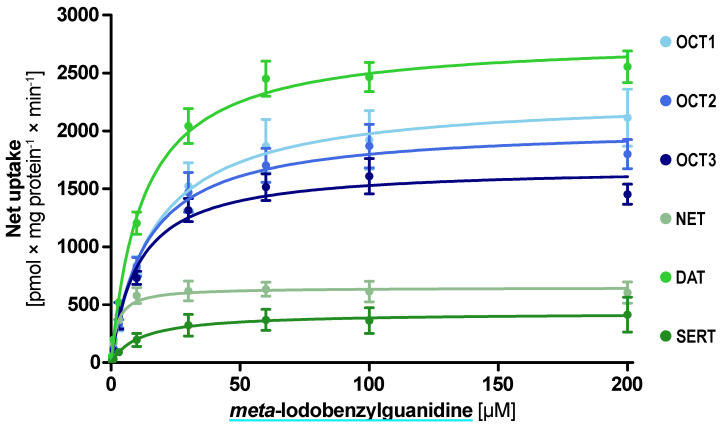
Concentration-dependent uptake of *meta*-iodobenzylguanidine by the investigated transporters. Transport is presented as net uptake with the mean ± SEM of three independent experiments.

The kinetic analyses revealed low K_m_ uptake of mIBG by the aforementioned five transporters and uptake with a higher K_m_ by SERT (Figure 5). The lowest K_m_ and highest intrinsic clearance were found for NET with a K_m_ of 2.3 µM and Cl_int_ of 284 mL × g protein^−1^ × min^−1^ (Table 1). Interestingly, DAT was the transporter with the highest v_max_ with 2800 pmol × mg protein^−1^ × min^−1^.

In addition to the phenylethylamines, the second major group of investigated compounds belonged to the class of substituted tryptamines. With minor exceptions, for the tryptamine-based compounds, the same structure–activity relationships were observed, as already described for the phenethylamine compounds above. Here, non-branched *N*-methylation reduced MAT uptake ratios if at all then only slightly, while OCT1 uptake was increased especially for *N*-methyl serotonin (Figure S6). 5-hydroxylation of the tryptamine-backbone greatly increased transport by MATs and to a minor extend also transport by OCT1. In line with the previous observations on *O*-methylations in phenylethylamines, MATs almost completely lacked the ability for transport of 5-methoxy tryptamine. 

The here formulated structure–activity relationships for the MATs were based on a single-concentration screening. To further demonstrate that distinct molecular features of the compound are required for efficient MAT transport, we finally compared concentration-dependently the uptake of the endogenous substrates for each MAT with the uptake of the respective parent compounds (phenylethylamine for NET as well as DAT and tryptamine for SERT). All MATs showed low K_m_ uptake for their respective neurotransmitter (Figure S7, Table S4) whereas the uptake the respective precursor was characterized by a low transport capacity and a high K_m_. These findings further highlight that although the unsubstituted parent compounds are transported as well, in particular, the backbone hydroxylations of phenylethylamine and tryptamine are critical for efficient transport by NET, DAT, and SERT. 

## 3. Discussion

In the last decades, MATs have been extensively characterized regarding their inhibition, but much less is known about their substrate spectrum. Polyspecific OCTs also transport monoamine neurotransmitters and numerous drugs acting as agonists or antagonists at monoaminergic receptors. Increasing evidence that OCTs and MATs cooperate in monoamine clearance during neuronal transmission [18,19,20,21,37,44] suggests a need for defining the functional specificity and overlap of both transporter families. 

To start with the most general finding, our data show that the substrate spectrum of MATs is larger than previously assumed but at the same time characterized by a low chemical diversity. Furthermore, our data show that MATs efficiently facilitate the uptake of numerous analogs of their endogenous substrates. Our results further substantiate that MAT transport is limited by the molar mass of the ligand, which is in line with insights from mutagenesis [45] and crystallography studies [46,47]. However, the molar mass is neither the only criterion nor are size limitations so strict that only compounds in the size of neurotransmitters are MAT substrates (as seen with frovatriptan for SERT, Figure S2). In addition to size, distinct molecular features are required for efficient MAT transport; especially hydroxylations at the phenylethylamine and tryptamine backbone facilitate MAT transport. This has previously been reported for DAT substrates [48] and in visionary studies on uptake_1_ even as early as in the 1960s (albeit with indirect measurements and of course without overexpression) [26]. We also observed that substitutions at the primary nitrogen of the phenylethylamine and tryptamine backbone decreased MAT transport (Figure 4 and Figure S4), this is in line with the historic findings using indirect methods and primary cells [26]. This also explains the low SERT uptake ratios of the recently shown hallucinogens dimethyl- and diethyltryptamine [43]. Interestingly and in contrast to the results in MATs, these substitutions at the primary amine improved transport via OCT1 in several cases.

A rather unexpected finding was SERT showing moderate to high transport ratios for frovatriptan. As frovatriptan is larger than serotonin, these results highlight that the size cut-off for MAT substrates is not to be understood as a strict border a few Daltons higher than the molar mass of the transporters’ name-giving neurotransmitters. The main structural differences of frovatriptan and serotonin are the 5′-amide replacing the hydroxy moiety of serotonin, the third ring structure, and the secondary amino group. Based on our observations, the single methyl substituent at the nitrogen is well tolerated, however, for the other modifications we lack the molecular pairs for direct comparison. From a chemical point of view, the additional amide can engage in similar molecular interactions as the replaced hydroxy group, functioning as hydrogen-bond donor and acceptor as well. The ring closure, that makes frovatriptan much less adaptable, suggests that SERT provides some extra space in its respective binding pockets allowing for chemical modifications of known ligands. From this data, it might be speculated that there is a particular accumulation of frovatriptan into thrombocytes (known to highly express SERT) and in presynaptic serotoninergic neurons. However, we did not find any data on whether this was confirmed in human beings or whether this has any clinical implications. Nonetheless, one might still speculate that high accumulation may result in toxicity or might interfere with other cellular processes. All other triptans tested were not transported by SERT.

While exceeding typical size limitations does not exclude transport with certainty, as discussed above, undercutting typical size limitations does not guarantee MAT transport either. Many compounds matching size limitations are not efficiently transported by MATs. Exemplarily, phenylethylamine (PEA) shows low transport ratios as well as a high K_m_, low-capacity kinetics for MATs. This is not inconsistent with the fact that PEA shows NET/DAT inhibition with IC50 values in the low micromolar range [49]. PEA might interact with the transporter but could nevertheless lack the molecular features for efficient transporter-mediated translocation.

Many of the compounds that were efficiently transported by MATs and also by OCT2 belong to the group of trace amines including tyramine, octopamine, synephrine, and tryptamine [50]. These compounds are mostly of endogenous and/or microbial origin and act via trace amine-associated receptors (TAARs), intracellular G-protein coupled receptors [51], which modulate for instance monoaminergic neurotransmission [52]. Transporters are discussed to be involved in the regulation of synaptic levels of trace amines and OCT2 has been recently identified as high-affinity tyramine transporter [53]. Among all transporters tested in our study, OCT2 showed the broadest substrate spectrum with medium to high uptake ratios for almost all low molar mass amines. Interestingly, the intracellular target [50,54] of *p*-tyramine, TAAR1, is also characterized by a rather broad substrate specificity [55,56]. One could speculate that neuronal expressed OCT2 and TAAR1 complement each other in regulating neuronal levels and action of trace amines. Uptake by neuronal OCTs or MATs into monoaminergic neurons might also have additional consequences. Given the structural similarity of trace amines and monoamine neurotransmitters, the accumulation of trace amines might interfere with vesicular neurotransmitter storage and enzyme-mediated degradation. Among trace amines, transporters also facilitate the uptake of the neurotoxic compounds salsolinol [57]. Many of these compounds are not only endogenous intermediates but also occur naturally in several plants or microbial species and might therefore be taken up by nutrition (Table 2).

Uptake by extraneuronal (e.g., hepatic and renal) expressed OCTs might be a critical step in the elimination of these compounds. Exemplarily, the OCT2-mediated uptake and excretion of *p*-tyramine via the kidney, but also p-tyramine transport by NET, DAT, and OCT3 (Figure 1), might all contribute to the observation that interindividual variation of tyramine pharmacokinetics and pharmacodynamics is not determined at all by the OCT1 genotype [75].

MAT and OCT transport has rarely been investigated comparatively, to the regret of those in search of specific MAT tracers. As shown here, with mIBG, comparative data using the same assay conditions, reinforces the clinically supported observation, that tracers can have little specificity not only between certain MATs, but also between MATs and OCTs and potentially other cation transporters as well. Undesired tracer uptake by organic cation transporters causes several issues. In neuroendocrine cancer treatment, unwanted OCT1-mediated uptake into the liver explains occasionally observed liver toxicity indicated by increased serum transaminases [76]. In mIBG-based cardiac imaging, liver uptake causes unwanted background noise where the left liver lobe is in close proximity to the heart. Furthermore, due to SERT expression in platelets and megakaryocytes and again due to mIBG lacking NET selectivity, mIBG therapy in neuroendocrine cancer is often limited by myelosuppression with low platelet counts [77]. In addition, OCT3-mediated mIBG uptake into heart tissue [78,79] can feign neuroendocrine metastases. 

A strategy for devising an OCT-specific substrate tracer without transport by MATs is relatively straightforward: Our data here reveals, as mostly to expect, clear signs of a size cutoff for transport in MATs that is not matched by a similarly strict cutoff in OCTs.

The opposite goal of devising MAT-specific substrate tracers without transport by OCTs, from a current clinical standpoint the more interesting optimization, is unfortunately the harder one and our dataset provides unfortunately little clue on a solution. An obvious observation in our data is OCT2 being the strongest competitor for MATs. Ring hydroxylations in *meta* and/or *para* position shifted the specificity towards MATs and away from OCTs. This was not just true for the obvious cases, the neurotransmitters themselves, but also, e.g., for tyramine, when compared with phenylethylamine, and for octopamine and norphenylephrine, when compared with bisnorephedrine. This raises the question of whether hydrophilic ring substitutions are detrimental for OCT transport or simply beneficial to MAT transport. The frequent occurrence of ring hydroxylations in good OCT substrates such as pirbuterol, salbutamol, and salsolinol indicate that OCTs can at least deal with it, though it might not benefit as much. With a strict size cut-off for MAT transport, there are of course little degrees of freedom remaining for medicinal chemistry approaches to make proper MAT substrates poorer OCT substrates. In that regard, it is relieving to see that at least for SERT, in frovatriptan, an exception for the size cut-off could be identified. Further examination of substrate candidates, which claim the middle ground between neurotransmitters and too large compounds, might provide hints for MAT-specific tracers. The use of co-administered OCT inhibitors to improve MAT/OCT tracer selectivity, which has also been brought up [80], is no viable alternative to finding a more selective substrate. Although OCT inhibition is a reasonable approach, it might create new problems given the prominent role of OCTs in the pharmacokinetics of many cationic drugs [14,81]. With the polyspecificity of OCTs, finding a specific inhibitor might be already a challenging goal. 

In search of MAT-specific tracers, one worthwhile future objective for OCT research could be the search for small cationic OCT non-substrates. This approach might indeed be more challenging with a polyspecific transporter than to find additional substrates.

## 4. Materials and Methods

### 4.1. Test Compounds

Compounds with a structure very similar to the known neurotransmitter substrates of MATs were selected for this study. This resulted in 36 substances with whole or partial phenethylamine or tryptamine backbone and with a molar mass between 121 Da and 240 Da. To possibly identify molecular size boundaries for MATs, compounds, still, phenethylamine- or tryptamine-based, but with larger substitutions were identified within the groups of monoaminergic receptor agonists (α- and β-adrenoceptor agonists as well as serotonin receptor agonists). These larger compounds had molar mass from 243 Da up to 383 Da. Additional less closely related compounds were selected based because of clinical significance, e.g., mIBG.

Test compounds were bought from Sigma-Aldrich (Taufkirchen, Germany), Toronto Research Chemicals (Toronto, ON, Canada), Santa Cruz Biotechnology (Darmstadt, Germany), LGC Standards (Luckenwalde, Germany), and Wako Chemicals (Neuss, Germany). A complete list of all compounds used in this study and their manufacturers is provided in Supplementary Table S5.

### 4.2. In Vitro Transport Experiments

Transporter function was investigated via an in vitro uptake assay using HEK293 cells stably transfected to overexpress the specific transporter. Cell lines were generated using the Flp-In system as described previously [11,43,82,83], except for hOCT3 overexpressing cells which were a kind gift from Drs Koepsell and Gorbulev (University of Würzburg, Würzburg, Germany). Cells were regularly cultivated in DMEM with 10% (*v*/*v*) FCS and 1% (*v*/*v*) penicillin/streptomycin and kept in culture for up to 30 passages.

For cellular uptake assays, 300,000 cells were plated per well in poly-D-lysine coated 24-well plates two days ahead of the experiment. The transport experiments were carried out at 37 °C and cells were washed once with 1 mL pre-warmed HBSS+ (10 mM HEPES in Hank’s balanced salt solution, pH 7.4; Thermo Fisher Scientific, Darmstadt, Germany) prior to incubation. Cells were then incubated with 2.5 μM test compound in 37 °C HBSS+ for exactly two minutes and incubation was stopped by adding 1 mL of ice-cold HBSS+ (epinephrine and norepinephrine uptake ratios were determined with an incubation period of 5 min). Finally, cells were washed twice with ice-cold HBSS+ and lysed using 80% acetonitrile (LGC Standards, Wesel, Germany) containing an appropriate internal standard for high-performance liquid chromatography coupled to mass spectrometry (HPLC-MS/MS), internal standards are provided in Table S5. For concentration-dependent uptake analyses, cells were incubated with increasing drug concentrations, and additionally, a standard curve of known compound concentrations was prepared for eventual quantification. Additionally, per cell line, the cells in one well were lysed using RIPA buffer and total protein content was quantified in a bicinchoninic acid assay [84]. Total protein content was then used for normalization of cellular uptake to the density of seeded cells.

### 4.3. Concentration Analyses

Intracellular drug concentrations were quantified by HPLC-MS/MS analysis using a Shimadzu Nexera HPLC system with a SIL-30AC autosampler, a CTO-20AC column oven, an LC-30AD pump, and a CBM-20A controller (Shimadzu, Kyoto, Japan). Compounds were separated on a Brownlee SPP RP-Amide column (4.6 × 100 mm inner dimension with 2.7 µm particle size) with a C18 pre-column. Reverse-phase chromatography was carried out with an aqueous mobile phase containing 0.1% (*v*/*v*) formic acid and either 3, 8, or 20% (*v*/*v*) organic additive (acetonitrile:methanol (6:1)) with a flow rate of 0.3 mL min^−1^ and an oven temperature of 40 °C. Detection was carried out with an API 4000 tandem mass spectrometer (AB SCIEX, Darmstadt, Germany) operating in MRM mode. Analyte peaks were integrated and quantified using the Analyst software (Version 1.6.2, AB SCIEX, Darmstadt, Germany). All analytes, corresponding internal standards, HPLC mobile phase composition, and MS detection parameters are listed in Supplementary Table S5.

### 4.4. Calculations

Transporter-mediated uptake was quantified via uptake ratios which are calculated as the quotient of cellular uptake in transporter-overexpressing cells by uptake of empty-vector transfected control cells each normalized by total protein content in a control well. For concentration-dependent analyses, transporter-mediated net transport was calculated by subtracting cellular uptake into empty-vector control cells from cellular uptake into the transporter-overexpressing cells. Kinetic parameters were obtained by non-linear regression analysis according to the Michaelis–Menten (v = v_max_ × [S]/(K_m_ + [S]) equation using GraphPad Prism (Version 5.01 for Windows, GraphPad Software, La Jolla, CA, USA). V_max_ is the maximum transport velocity and Km is defined as the compound concentration required to reach half of v_max_. Intrinsic clearance Cl_int_ was then calculated as the quotient of v_max_ and K_m_.

## Figures and Tables

**Table 1:** Uptake characteristics of *meta*-iodobenzylguanidine.

Transporter	Uptake Ratio [x-Fold over EV Control]	K_m_ ± SEM [µM]	V_max_ ± SEM [pmol × mg Protein^−1^ × min^−1^]	Cl_int_ ± SEM [mL × g Protein^−1^ × min^−1^]
OCT1	11.4 ± 2.2	16.6 ± 3.7	2301 ± 129	138 ± 38.5
OCT2	14.7 ± 1.6	13.3 ± 2.4	2039 ± 86.9	152 ± 34.1
OCT3	12.0 ± 1.0	10.5 ± 1.9	1689 ± 68.0	161 ± 35.7
NET	11.3 ± 2.1	2.27 ± 0.57	648 ± 28.2	284.6 ± 84.2
DAT	15.1 ± 3.0	12.2 ± 1.46	2800 ± 77.2	229 ± 33.8
SERT	4.3 ± 1.0	11.2 ± 6.2	429 ± 53.4	38.2 ± 25.9

**Table 2:** Typical origin of trace amine-like compounds investigated in this study.

Name	Endogenous	Microbiom	Nutrition	Plants	Example and References
3-Methoxy *p*-tyramine	x			x	Dopamine metabolite Cactus [58]
Bisnorephedrine	x				Trace amine neuromodulator [59]
Deoxyepinephrine				x	*Acacia* species [60]
Halostachine				x	Perennial ryegrass, tall fescue [61]
Hordenine			x	x	Barley, beer [62] *Acacia* species [60]
*N*-Methyl phenylethylamine	x			x	Trace amine neuromodulator *Acacia* species [63]
*N-*Methyl serotonin				x	Black cohosh [64]
*N-*Methyl tryptamine	x			x	Citrus plants [65]
*N*-Methyl *p*-tyramine	x			x	*Acacica* speciest [60]
Octopamine	x		x	x	Trace amine neuromodulator [66] Citrus herbs [67]
Phenylethylamine	x		x		Cheese [68]
Salsolinol	x		x	x	Dopamine intermediate [57] Cocao, chochlate [69]
Synephrine			x	x	Various *Citrus* trees [70]
Tryptamine	x	x	x		Trace amine neuromodulator [71] Commensual bacteria [72], Cheese [68]
*m*-Tyramine	x				Trace amine neuromodulator [73]
*p*-Tyramine	x	x	x		*Enterococcus* species [74], cheese [68] Citrus herbs [67]

## Data Availability

The raw data supporting the conclusions of this article will be made available by the authors, without undue reservation.

## Supplementary Materials

The following are available online at https://www.mdpi.com/article/10.3390/ijms222312816/s1.
