# Supplementary material for: Overlap and Specificity in the Substrate Spectra of Human Monoamine Transporters and Organic Cation Transporters 1, 2, and 3
Source: Int J Mol Sci. 2021 Nov 26;22(23):12816. doi: 10.3390/ijms222312816 (PMC8657982; doi:10.3390/ijms222312816)
Supplement: Supplementary file 1 [file ijms-22-12816-s001.zip › FigureS1.pdf]

Figure S1: Molecule Structures and Ratio Plots

Phenylethylamines I

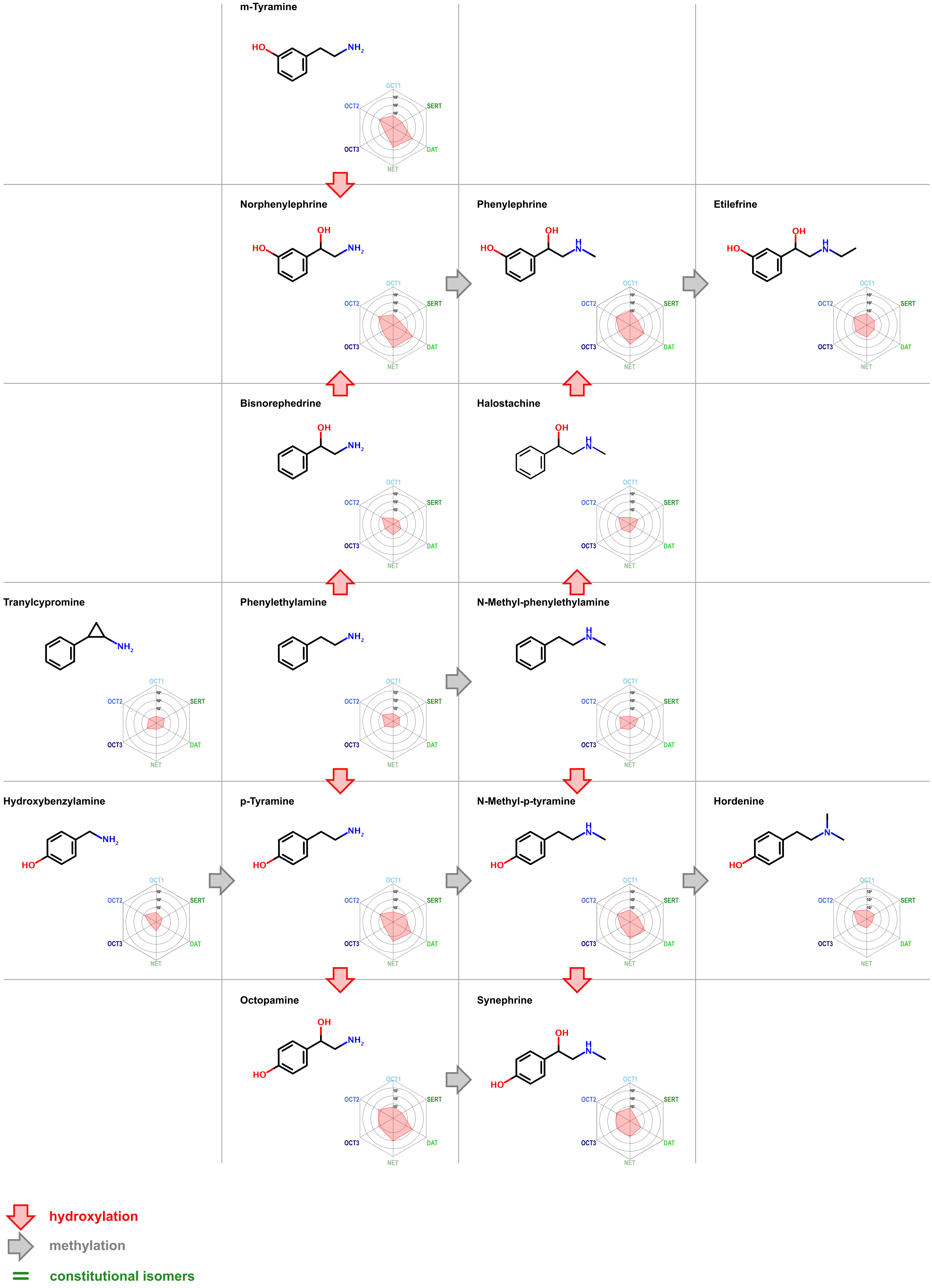

Phenylethylamines II

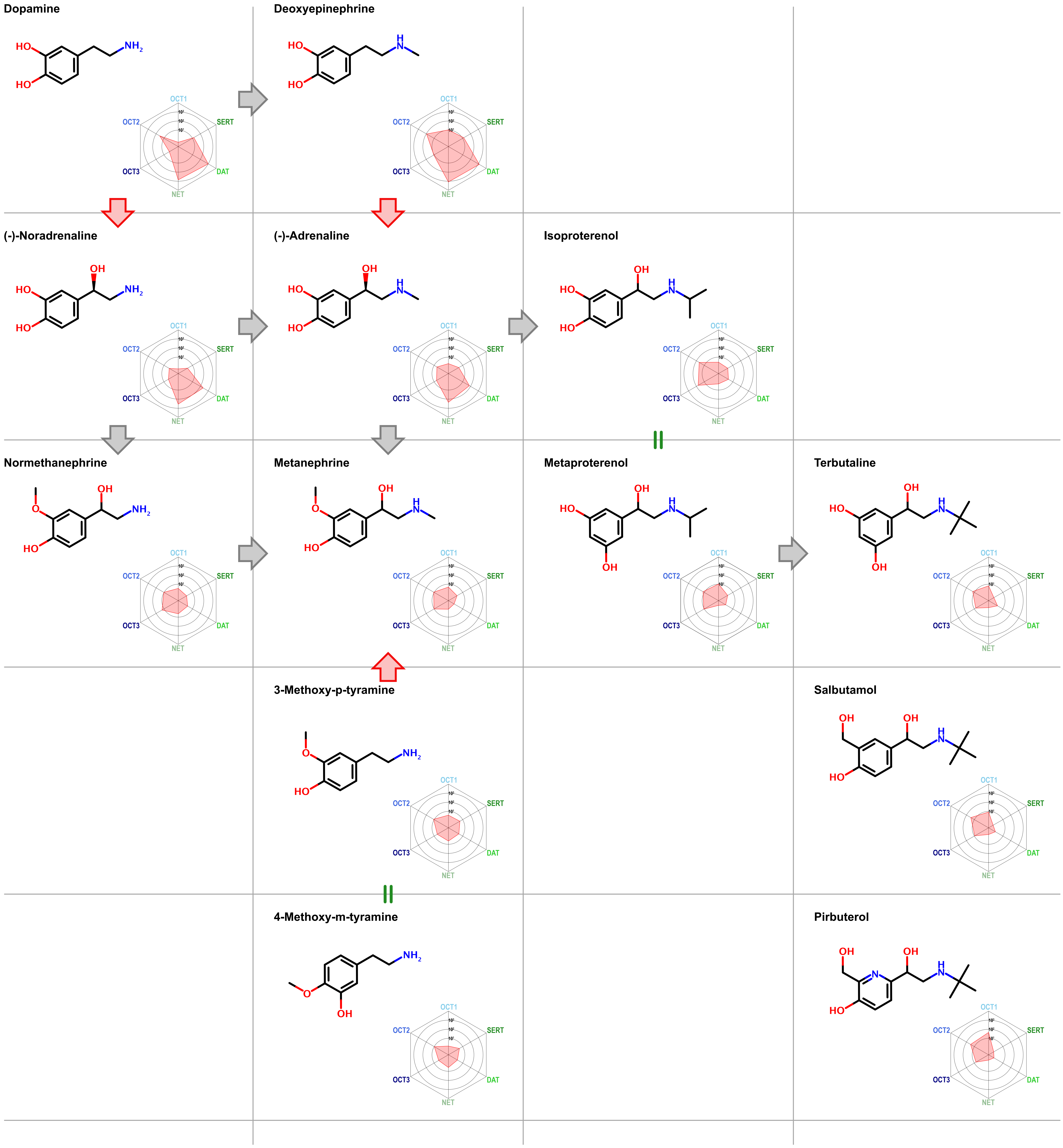

hydroxylation

methylation

constitutional isomers

Figure S1: Molecule Structures and Ratio Plots

Indoles

Tryptamine

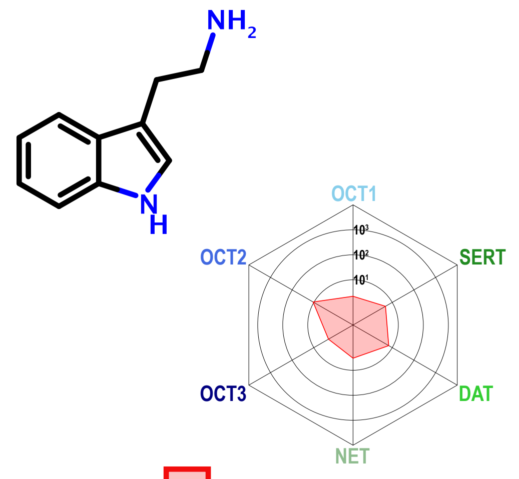

N-Methyltryptamine

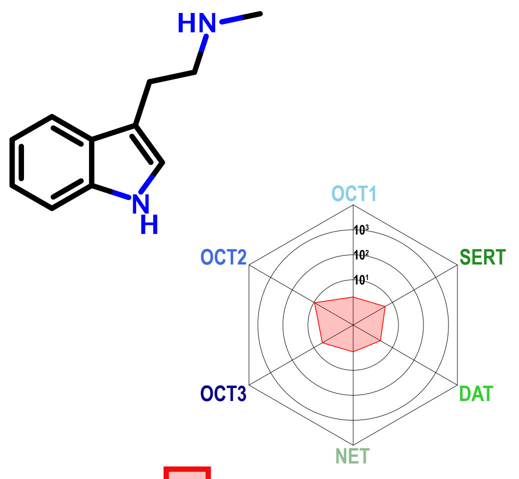

5-Methoxytryptamine

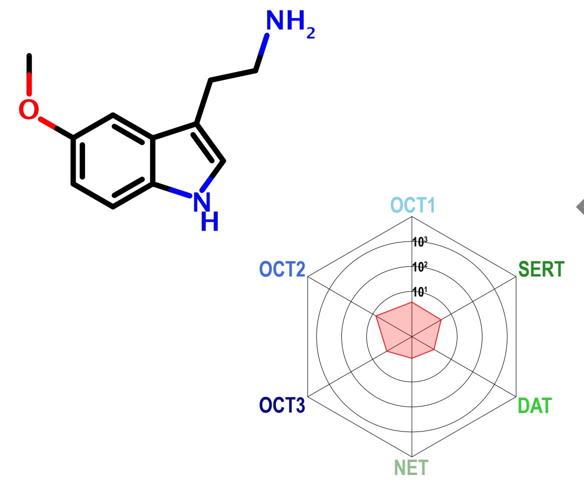

Serotonin (5-HT)

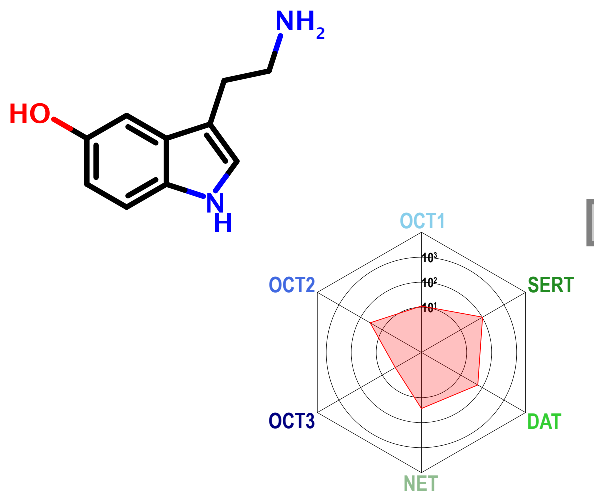

N-Methylserotonin

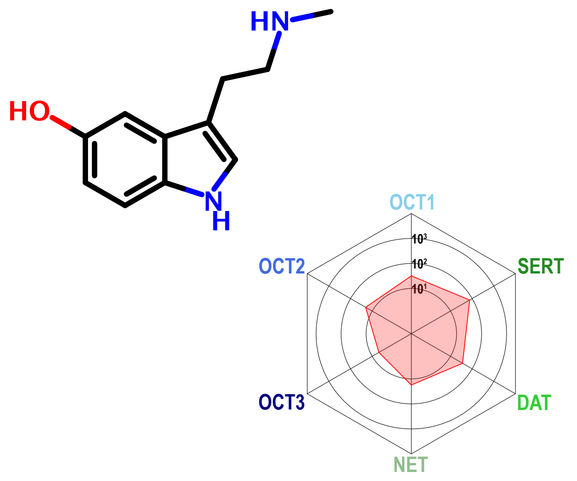

Eletriptan

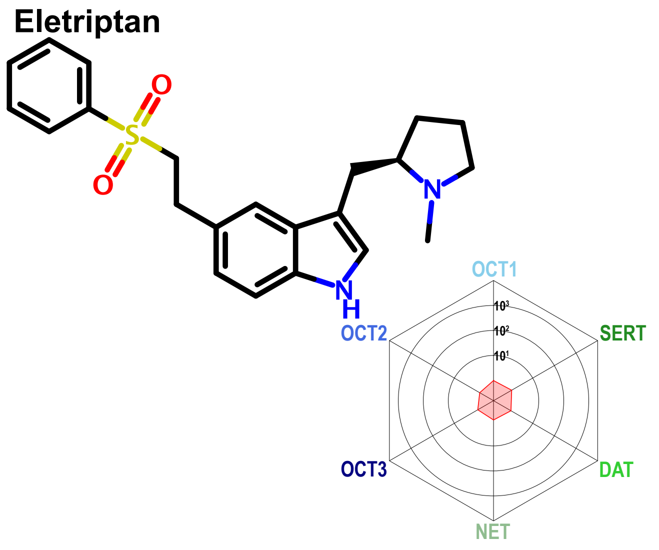

Frovatriptan

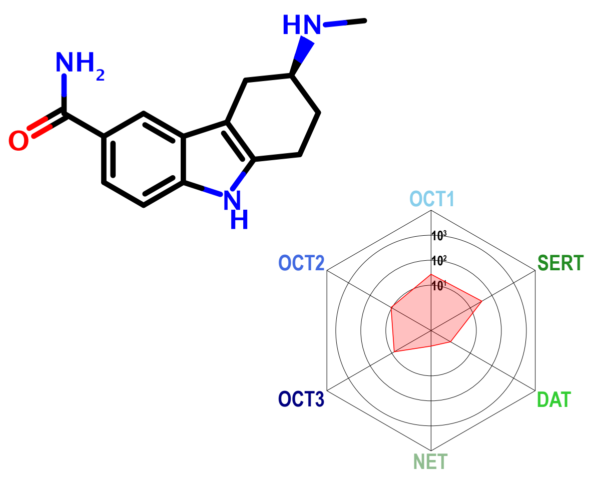

Rizatriptan

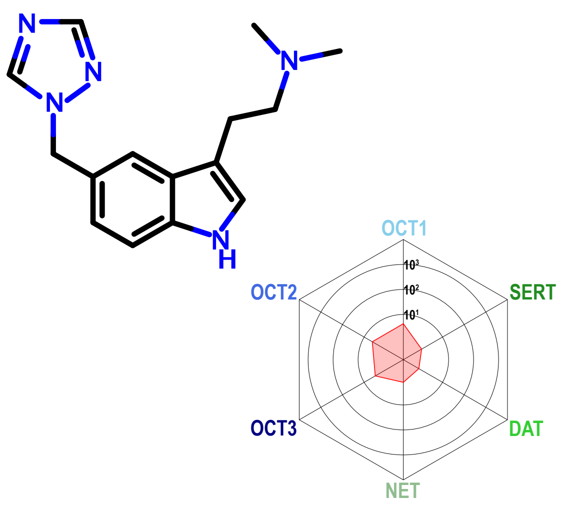

Sumatriptan

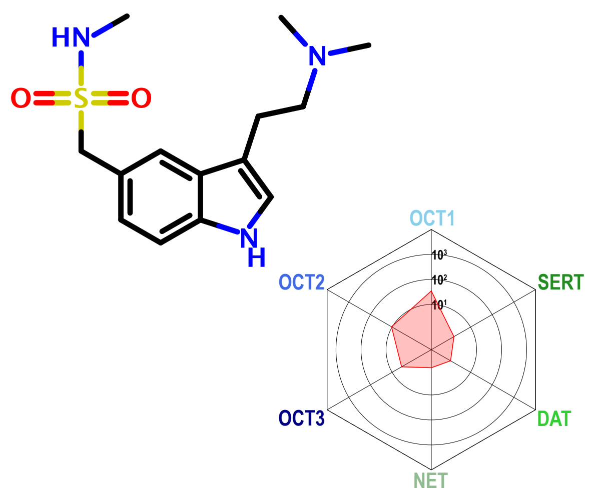

Zolmitriptan

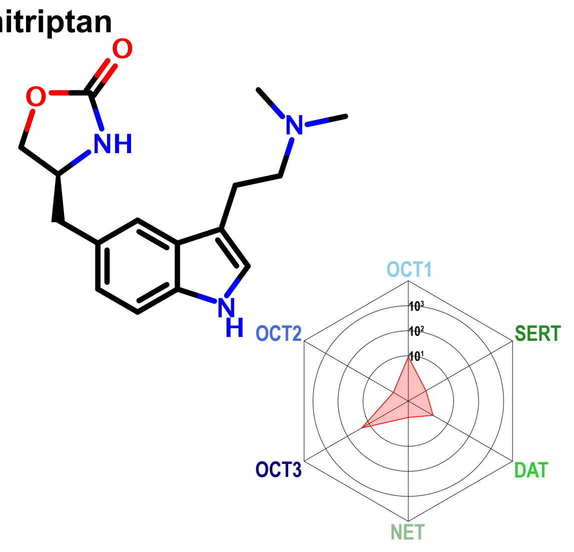

hydroxylation

methylation

constitutional isomers

Figure S1: Molecule Structures and Ratio Plots

Beta-adrenergic Agonists and Others

Dobutamine

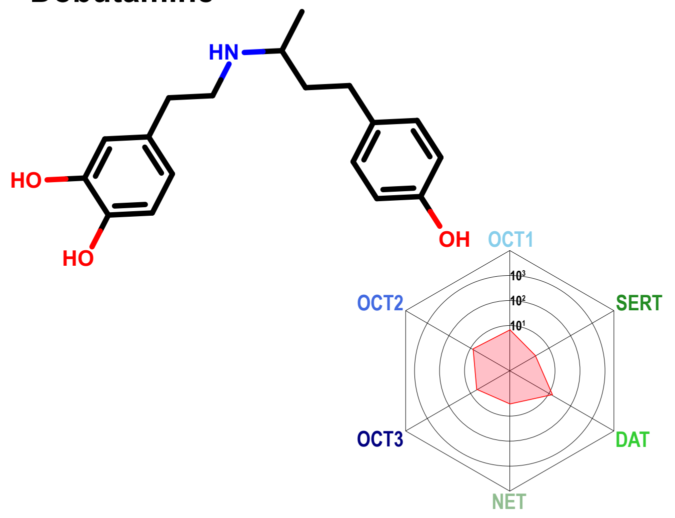

Fenoterol

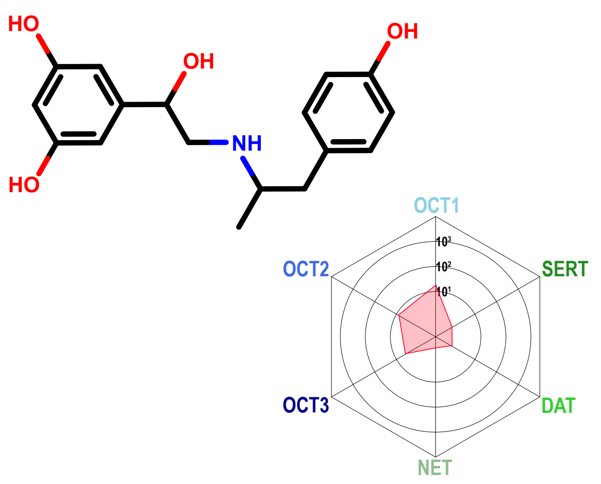

Formoterol

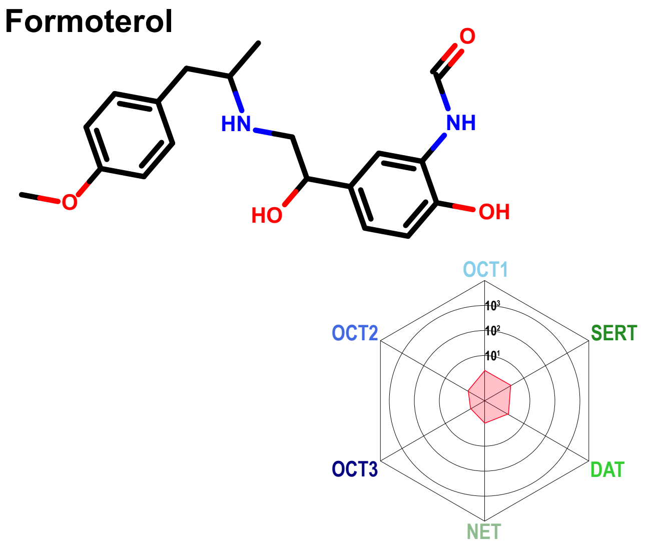

Ractopamine

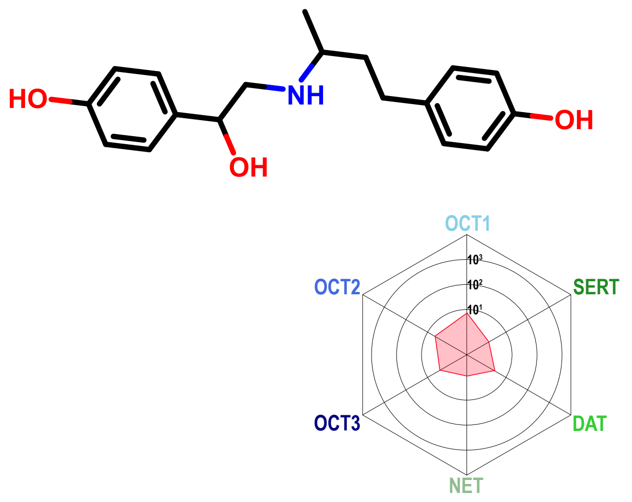

Clenbuterol

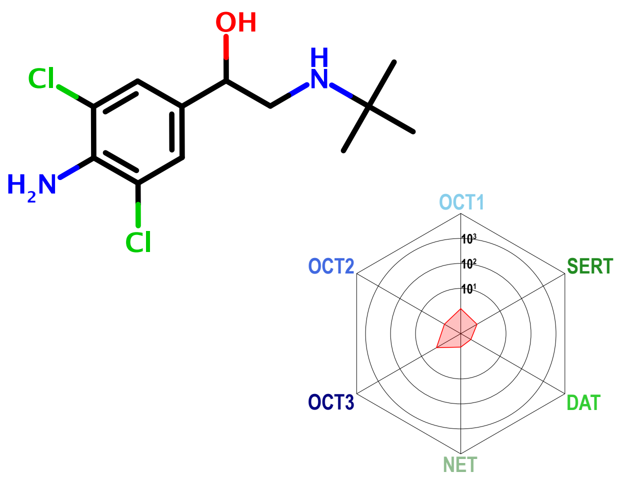

Ethambutol

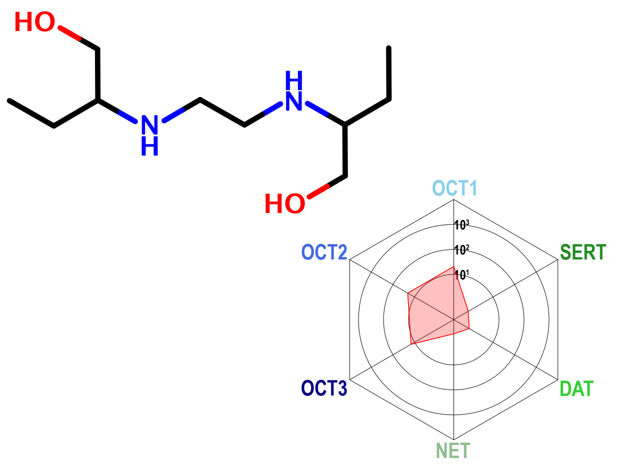

Metaiodobenzylguanidine

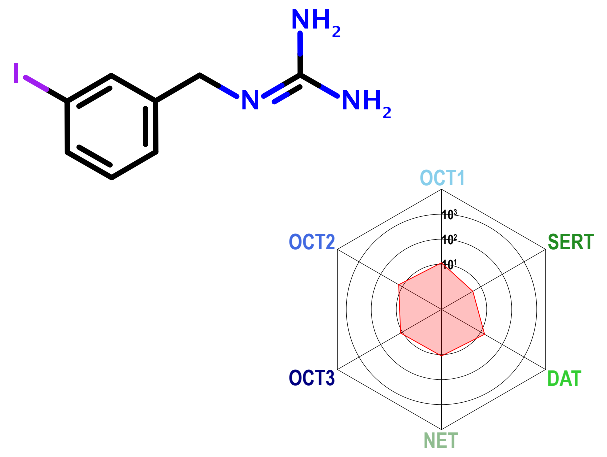

MPP⁺

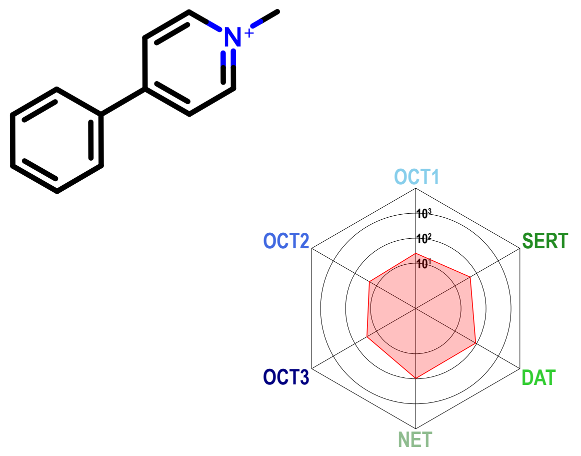

Salsolinol

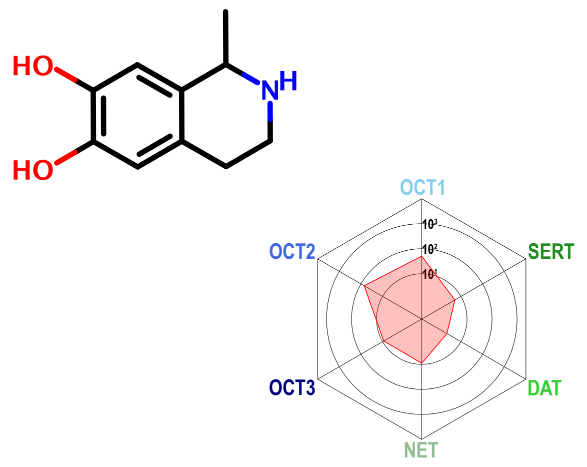

Sotalol

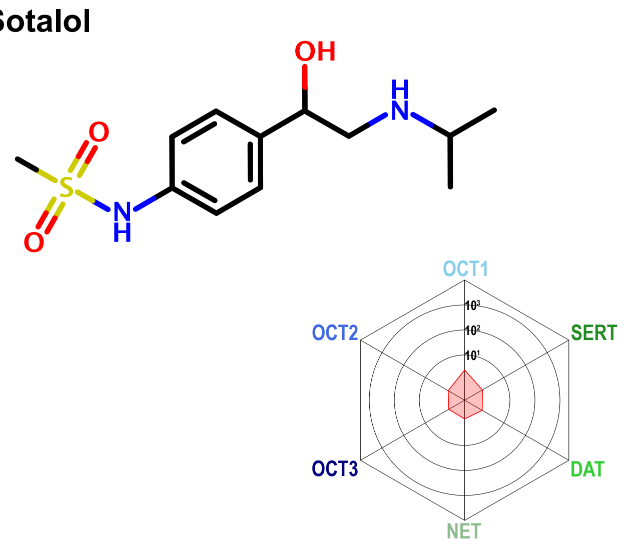

hydroxylation

methylation

= constitutional isomers
