# Supplementary material for: Overlap and Specificity in the Substrate Spectra of Human Monoamine Transporters and Organic Cation Transporters 1, 2, and 3
Source: Int J Mol Sci. 2021 Nov 26;22(23):12816. doi: 10.3390/ijms222312816 (PMC8657982; doi:10.3390/ijms222312816)

N-Methylation

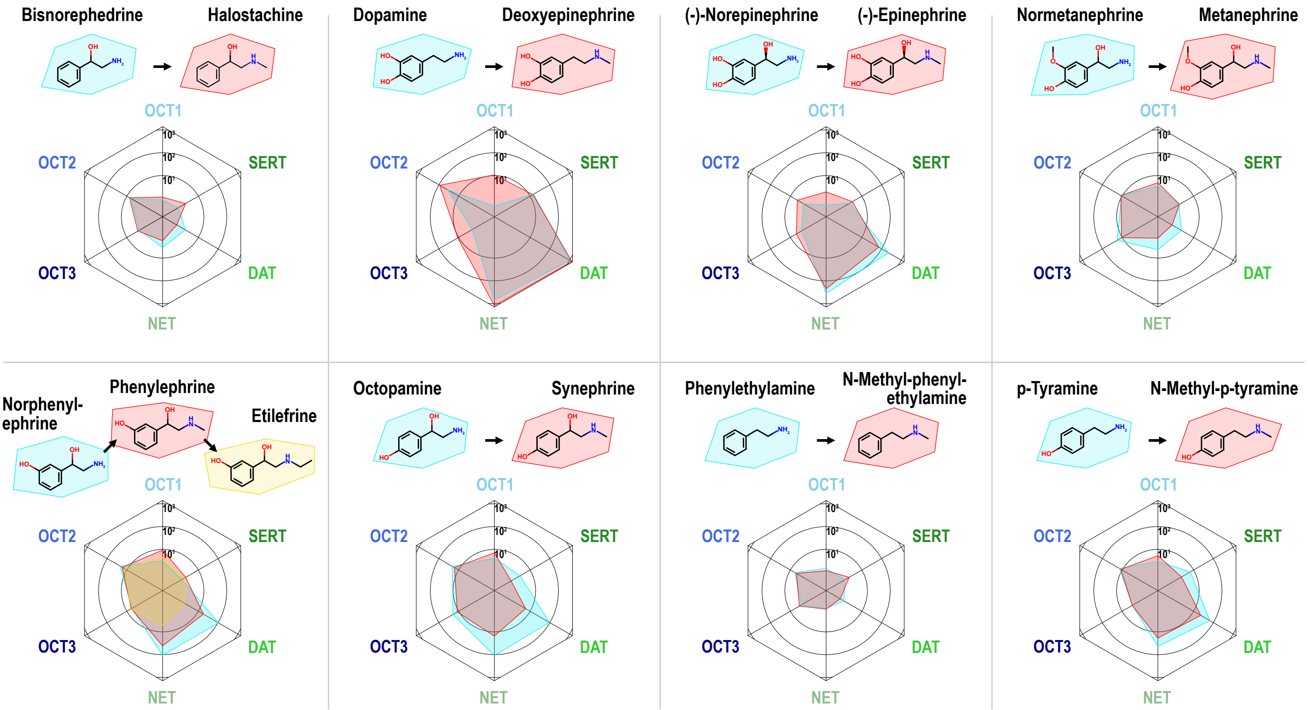

Meta-Hydroxylation

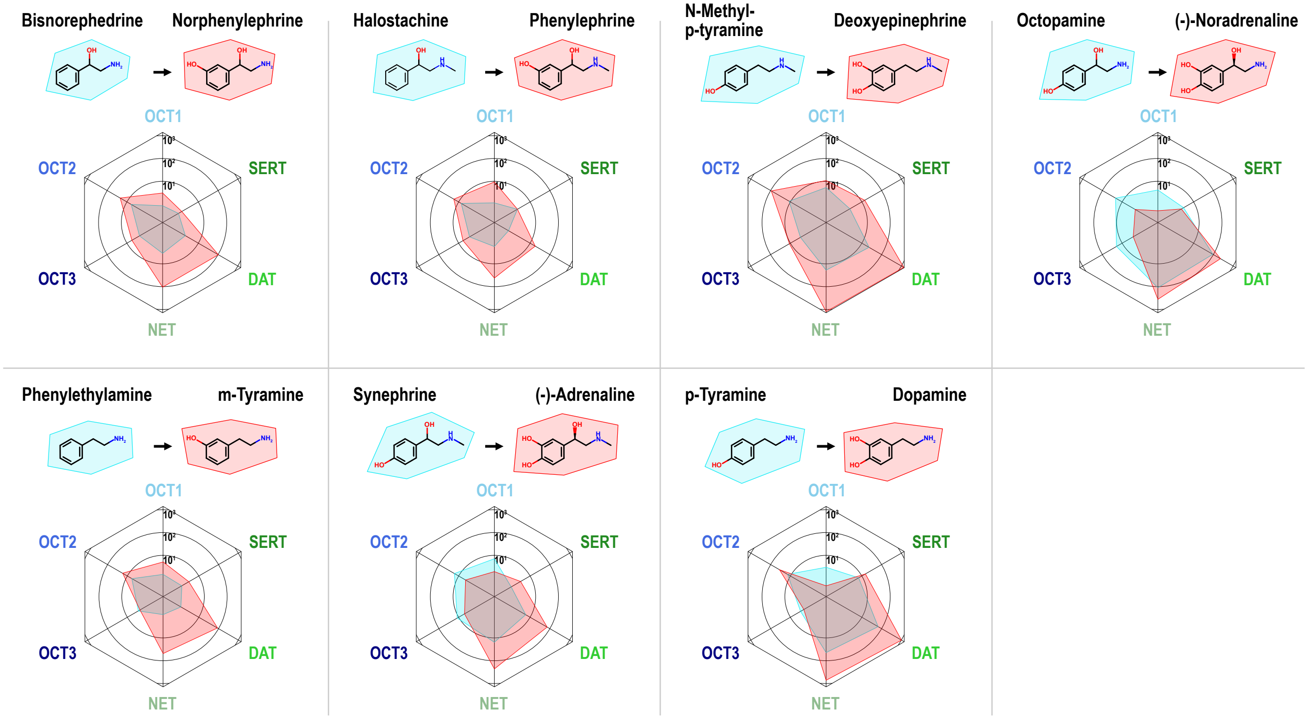

O-Methylation

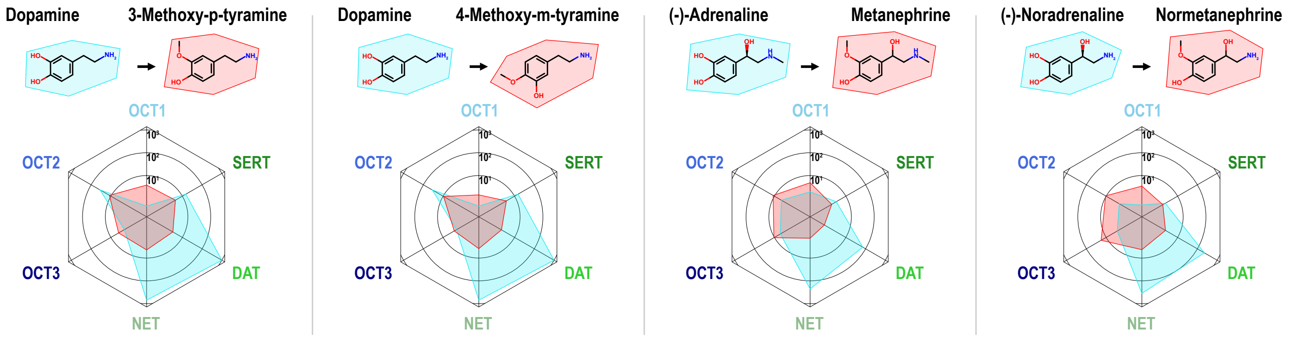

Para-Hydroxylation

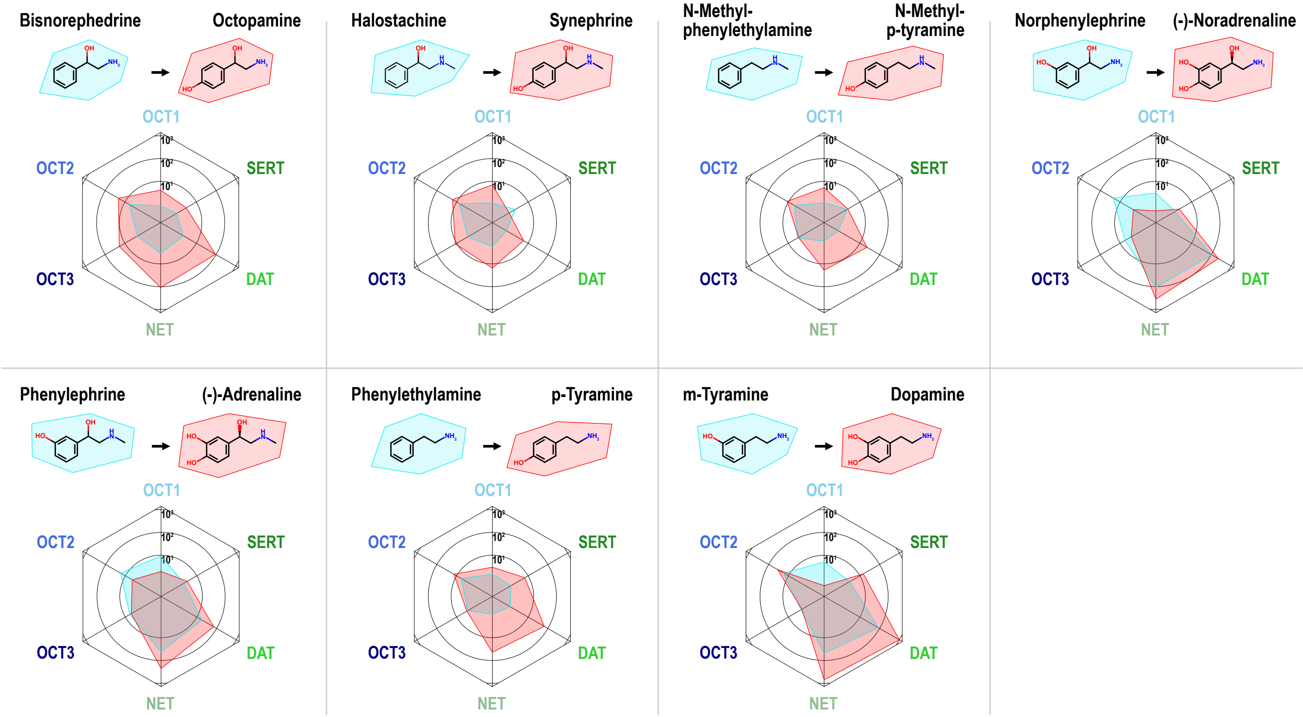

Beta-Hydroxylation

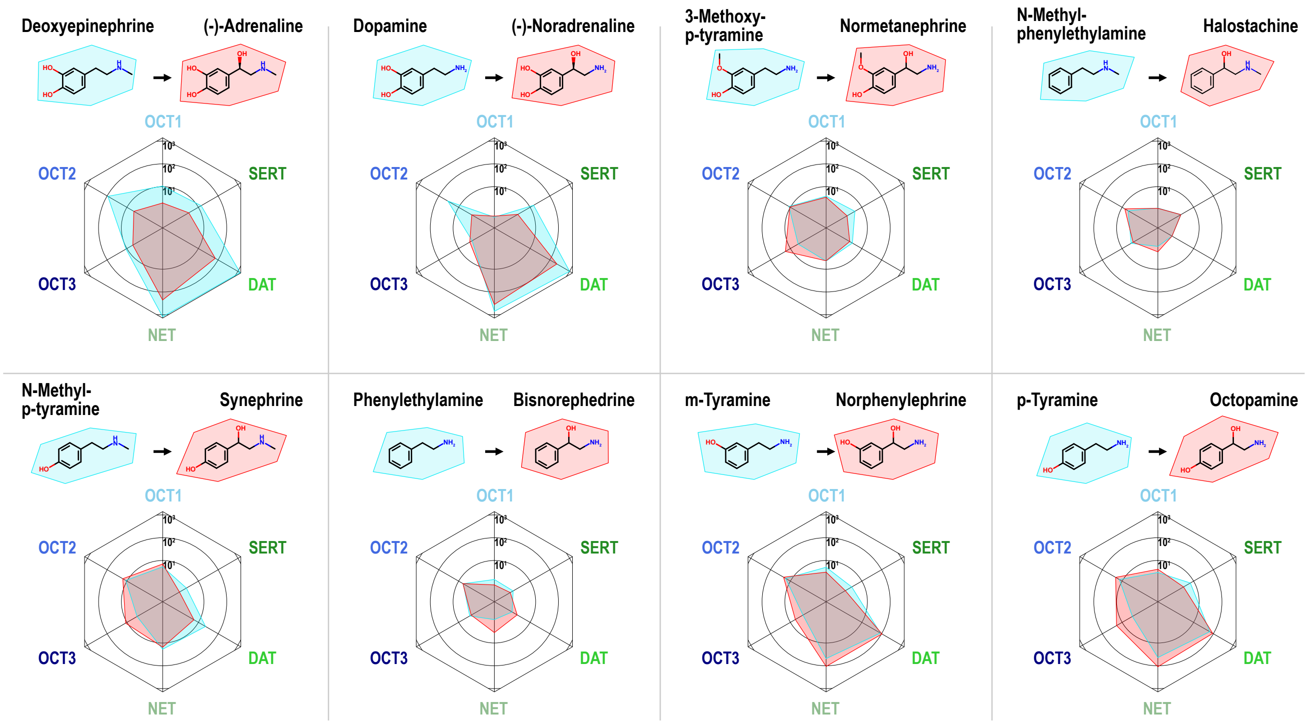

Supplement: Supplementary file 1 [file ijms-22-12816-s001.zip › FigureS5.pdf]
