# Supplementary material for: Overlap and Specificity in the Substrate Spectra of Human Monoamine Transporters and Organic Cation Transporters 1, 2, and 3
Source: Int J Mol Sci. 2021 Nov 26;22(23):12816. doi: 10.3390/ijms222312816 (PMC8657982; doi:10.3390/ijms222312816)
Supplement: Supplementary file 1 [file ijms-22-12816-s001.zip › Supporting Information.pdf]

Overlap and specificity in the Substrate Spectra of Human Monoamine Transporters and Organic Cation Transporters 1-3

Lukas Gebauer, Ole Jensen, Maria Neif, Jürgen Brockmöller, Christof Dücker

Institute of Clinical Pharmacology, University Medical Center Göttingen, D-37075 Göttingen, Germany

Supporting Information

Table of Contents

Figure S1: Molecule structures and comparative ratio plots (separate file);

Figure S2: Transporter-specificity plots, sequence identity and uptake correlation matrices;

Figure S3: Comparative uptake of frovatriptan and serotonin by SERT;

Figure S4: Concentration-dependent uptake of serotonin and *N*-methyl serotonin by MATs and OCTs. Bar plots and correlation of kinetic parameters with uptake ratios;

Figure S5: Matched molecular pair analyses of phenylethylamine-based compounds (separate file);

Figure S6: Matched molecular pair analyses of tryptamine-based compounds (separate file);

Figure S7: Concentration-dependent uptake of monoamine neurotransmitters and their precursor compounds by MATs;

Table S1: SMILES codes of investigated drugs and uptake ratios via investigated transporters (separate file);

Table S2: Kinetic parameters of frovatriptan, serotonin and *N*-methyl serotonin uptake by MATs and OCTs; Table S3: Manufacturers, HPLC mobile phases and mass spectrometry detection parameters of analytes and internal standards;

Table S3: Comparison of chemical descriptors for highly transported substrates (defined by a transport ratio >3) of OCTs and MATs;

Table S4: Kinetic parameters of phenylethylamine, tryptamine and endogenous monoamine neurotransmitter uptake by MATs;

Table S5: Manufacturers, HPLC mobile phases and mass spectrometry detection parameters of analytes and internal standards

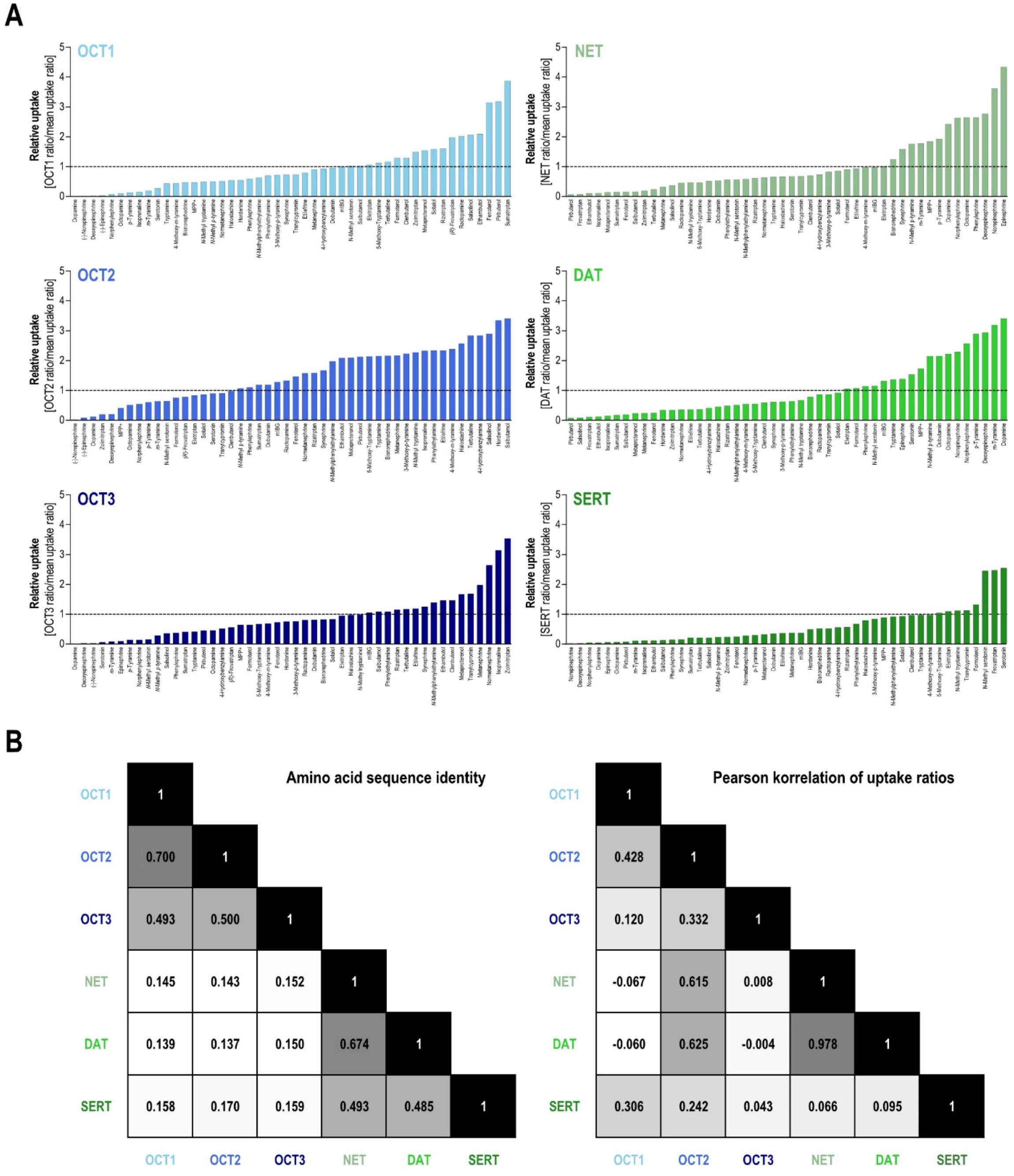

Figure S2: (A) Specificity of transporter uptake ratios. Specificity was calculated by dividing the uptake ratio for each transporter by the mean uptake ratio among all six transporters. **(B)** Amino acid sequence identity as determined by the “Multiple Sequence Comparison by Log-Expectation” (MUSCLE) provided by the European Bioinformatics Institute, Hinxton (https://www.ebi.ac.uk/), and correlation of uptake ratios for all six transporters.

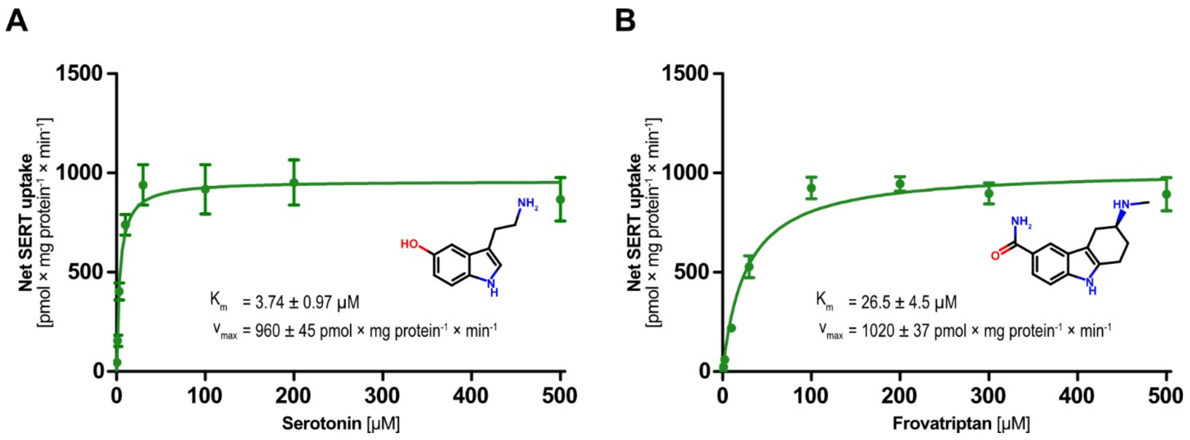

Figure S3 Uptake kinetics of serotonin **(A)** and frovatriptan **(B)** for SERT transport. Data is shown as mean ± SEM of at least three independent experiments.

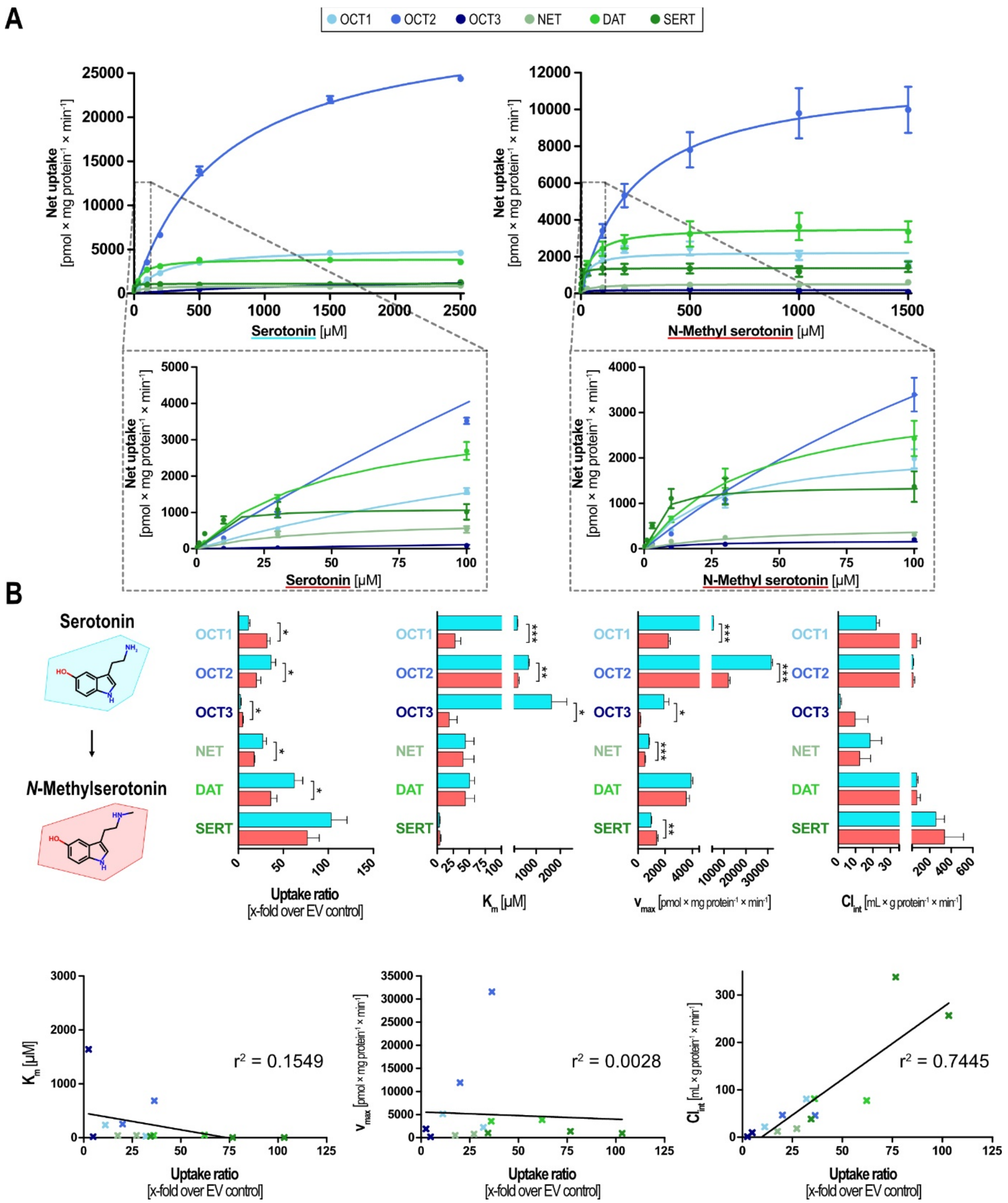

Figure S4: (A) Concentration-dependent uptake of serotonin and N-methyl serotonin by MATs and OCTs. Data is shown as mean ± SEM of at least three independent experiments. **(B)** Pairwise comparison of uptake ratio and transport kinetic characteristics. Differences were tested for statistical significance using Student's *t*-test with $^{*}p < 0.05$, $^{**}p < 0.01$, $^{***}p < 0.001$. Correlation of kinetic parameters K_m, v_{max} and Cl_{int} and uptake ratios. SERT-mediated transport of frovatriptan (Figure S3) was also included in this analysis.

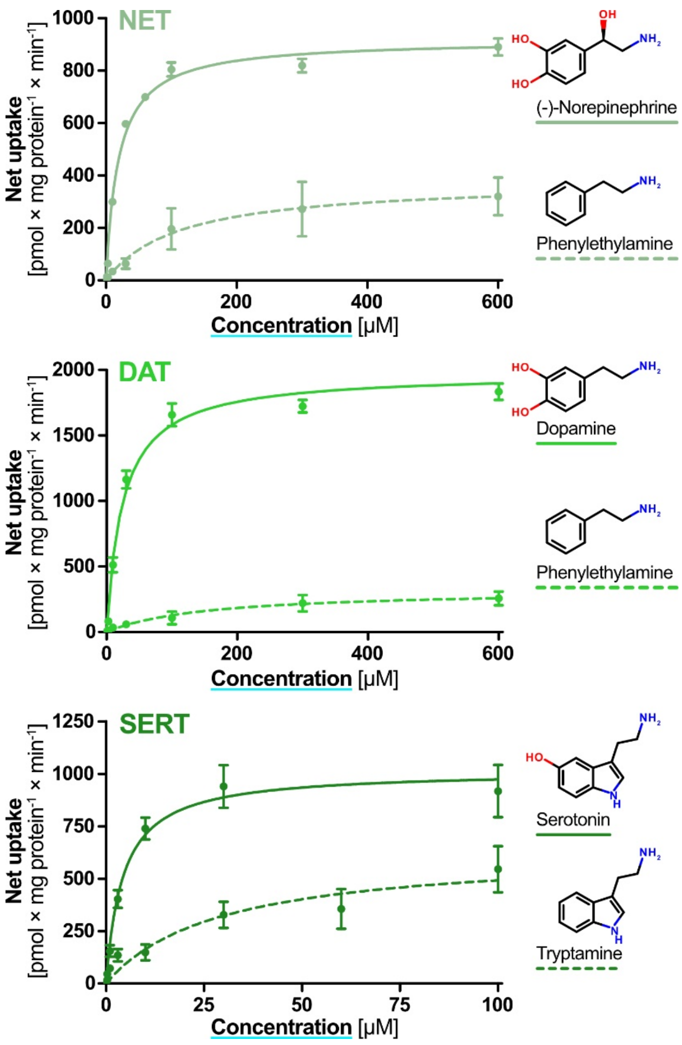

Figure S7 Concentration-dependent uptake of monoamine neurotransmitters and their precursor compounds by MATs NET, DAT and SERT. Data is shown as mean ± SEM of at least three independent experiments.

Table S2 Kinetic parameters of frovatriptan, serotonin and *N*-methyl serotonin uptake by MATs and OCTs

Transporter	Substrate	Uptake ratio [x-fold over EV control]	K_m ± SEM [µM]	V_{max} ± SEM [pmol × mg protein^{-1} × min^{-1}]	Cl_{int} ± SEM [mL × g protein^{-1} × min^{-1}]
OCT1	*N*-Methyl serotonin	31.9 ± 3.6	27.78 ± 9.19	2237 ± 135	80.52 ± 31.52
	Serotonin	11.2 ± 2.0	235.6 ± 15.50	5161 ± 95.0	21.91 ± 1.844
OCT2	*N*-Methyl serotonin	20.1 ± 5.0	253.9 ± 54.98	11918 ± 804	46.93 ± 13.33
	Serotonin	36.4 ± 5.1	685.6 ± 33.08	31565 ± 558	46.04 ± 3.04
OCT3	*N*-Methyl serotonin	4.9 ± 0.7	18.78 ± 12.42	181.7 ± 20.9	9.68 ± 7.51
	Serotonin	2.5 ± 0.7	1641 ± 630.2	1915 ± 366	1.17 ± 0.67
NET	*N*-Methyl serotonin	17.6 ± 0.6	40.57 ± 16.90	505.4 ± 41.1	81.24 ± 31.96
	Serotonin	27.2 ± 4.1	44.16 ± 13.33	809.4 ± 47.99	18.83 ± 6.62
DAT	*N*-Methyl serotonin	36.0 ± 6.8	43.82 ± 14.39	3560 ± 232	81.24 ± 31.96
	Serotonin	62.1 ± 9.4	50.50 ± 7.640	3908 ± 119.0	77.39 ± 14.06
SERT	Frovatriptan	34.3 ± 3.4	26.51 ± 4.52	1020 ± 36.5	38.48 ± 7.93
	N-Methyl serotonin	76.6 ± 13.1	4.065 ± 1.829	1373.0 ± 93.0	337.7 ± 174.8
	Serotonin	103.2 ± 17.5	3.736 ± 0.972	960 ± 44.9	256.9 ± 78.90

Table S3 Comparison of chemical descriptors for highly transported substrates (defined by a transport ratio >3) of OCTs and MATs. Data is provided as means (min;max), p-values are results from the overall group comparisons using the Kruskal-Wallis test. Charge and logD at pH 7.4 were calculated using the software cxcalc from ChemAxon, Budapest, Hungary, the remaining molecular descriptors were calculated using RDKit, RDKit: open-source cheminformatics software, https://www.rdkit.org/ (accessed Oct 7, 2021).

Descriptor	OCT1		OCT2		OCT3		NET		DAT		SERT		p-Value
Number of highly transported substrates (% of tested)	29/48		41/48		32/48		23/48		18/48		17/48		
logD at pH 7.4	-0.97	(-2.25 ; 1.16)	-1.01	(-2.25 ; 1.16)	-1.0	(-2.25 ; 1.16)	-1.12	(-1.82 ; 1.16)	-1.15	(-1.82 ; 1.16)	-1.15	(-1.74 ; 0.15)	0.831
Total polar surface area	60.2	(3.9 ; 93.0)	55.1	(3.9 ; 93.0)	56.1	(3.9 ; 93.0)	55.3	(3.9 ; 86.7)	55.0	(3.9 ; 86.7)	52.0	(3.9 ; 72.7)	0.637
Charge at pH 7.4	1.0	(1.0 ; 1.0)	1.0	(1.0 ; 1.0)	1.0	(1.0 ; 1.0)	1.0	(1.0 ; 1.0)	1.0	(1.0 ; 1.0)	1.0	(1.0 ; 1.0)	
Molar Mass	211.8	(137.2 ; 303.4)	192.1	(121.2 ; 303.4)	202.4	(133.2 ; 303.4)	175.7	(137.2 ; 301.4)	176.7	(137.2 ; 301.4)	177.9	(137.2 ; 275.1)	0.029
Heavy Atom Count	15	(10 ; 22)	13.7	(9 ; 22)	14.3	(10 ; 22)	12.4	(10 ; 22)	12.5	(10 ; 18)	12.5	(10 ; 18)	0.011
Heavy Atom Molar Mass	195.7	(126.1 ; 282.2)	177.2	(110.1 ; 282.2)	186.7	(122.1 ; 282.2)	162.9	(126.1 ; 278.2)	163.9	(126.1 ; 278.2)	165.2	(126.1 ; 265.0)	0.025
Number of H bond acceptors	3.1	(0 ; 5)	2.8	(0 ; 5)	2.9	(0 ; 5)	2.6	(0 ; 4)	2.5	(0 ; 4)	2.2	(0 ; 4)	0.116
Number of H bond donors	2.9	(0 ; 5)	2.7	(0 ; 5)	2.8	(0 ; 5)	2.7	(0 ; 4)	2.7	(0 ; 4)	2.4	(0 ; 4)	0.683
Number of Heteroatoms	3.6	(1 ; 6)	3.2	(1 ; 6)	3.4	(1 ; 6)	2.9	(1 ; 4)	2.9	(1 ; 4)	2.8	(1 ; 4)	0.055
Number of rotatable bonds	3.6	(0 ; 9)	3.2	(0 ; 9)	3.4	(0 ; 9)	2.6	(0 ; 7)	2.6	(1 ; 7)	2.2	(0 ; 3)	0.005
Number of valence electrons	81.2	(54 ; 118)	74.4	(48 ; 118)	78.2	(52 ; 118)	67.0	(54 ; 118)	66.7	(54 ; 118)	66.8	(54 ; 94)	0.008
Ring Count	1.5	(0 ; 3)	1.3	(0 ; 3)	1.4	(0 ; 3)	1.3	(1 ; 2)	1.3	(1 ; 2)	1.5	(1 ; 3)	0.701

Table S4 Kinetic parameters of phenylethylamine, tryptamine and endogenous monoamine neurotransmitter uptake by MATs

Transporter	Substrate	Uptake ratio [x-fold over EV control]	K_m ± SEM [μM]	V_{max} ± SEM [pmol × mg protein^{-1} × min^{-1}]	Cl_{int} ± SEM [mL × g protein^{-1} × min^{-1}]
NET	(-)-Norepinephrine	355 ± 67.6	19.2 ± 1.7	918 ± 18.2	47.8 ± 5.2
	Phenylethylamine	1.49 ± 0.23	113 ± 72.4	380.3 ± 78.4	3.4 ± 2.9
DAT	Dopamine	1000 ± 70.5	25.1 ± 3.0	1977 ± 46.2	78.9 ± 11.2
	Phenylethylamine	1.62 ± 0.45	156 ± 76.4	325 ± 54.1	2.1 ± 1.4
SERT	Serotonin	103.2 ± 17.5	3.736 ± 0.972	960 ± 44.9	256.9 ± 78.9
	Tryptamine	4.88 ± 1.30	29.0 ± 16.2	634.6 ± 129.5	22.0 ± 16.7

Table S5: Manufacturers, HPLC mobile phases and mass spectrometry detection parameters of analytes and internal standards

Compound	Manufacturer	RT [min]	Mass Q1 [Da]	Mass Q3 [Da]	DP [V]	CE [V]	CXP [V]	Internal standard
**3% organic additive** (96.9% H2O, 0.1 % formic acid, 2.6% acetonitrile, 0.4% methanol)								
Bisnorephedrine	Sigma-Aldrich	4.29	137.890	119.910 (63.910)	29	12 (37)	8 (12)	Buformin
Deoxyepinephrine	Toronto Research Chemicals	3.92	168.109	137.100 (91.100)	56	18 (38)	8 (16)	Buformin
Dopamine	Sigma-Aldrich	3.57	154.100	137.200 (91.000)	36	15 (33)	10 (8)	Buformin
Epinephrine	Sigma-Aldrich	3.17	184.100	135.000 (166.100)	36	15 (13)	8 (10)	Buformin
Ethambutol	Sigma-Aldrich	2.54	205.200	116.100 (55.100)	66	21 (45)	6 (10)	Buformin
Etilefrine	Santa-Cruz Biotechnology	4.46	182.124	164.000 (91.000)	51	17 (37)	10 (6)	Buformin
Halostachine	Sigma-Aldrich	4.63	152.100	134.000 (119.000)	41	13 (27)	8 (8)	Buformin
Hordenine	Sigma-Aldrich	4.49	166.140	121.000 (77.000)	51	21 (47)	8 (10)	Buformin
Isoprenaline	Sigma-Aldrich	4.02	212.200	151.900 (107.000)	41	23 (37)	10 (6)	Buformin
Metanephrine	Toronto Research Chemicals	3.51	198.100	165.000 (120.000)	41	25 (29)	10 (8)	Buformin
Metaproterenol	Sigma-Aldrich	4.63	212.130	152.000 (107.000)	56	23 (39)	10 (8)	Buformin
3-Methoxy *p*-tyramine	Toronto Research Chemicals	4.81	168.100	91.000 (119.000)	41	33 (25)	16 (8)	Buformin
4-Methoxy *m*-tyramine	Sigma-Aldrich	5.45	168.120	91.000 (118.900)	41	33 (25)	16 (8)	Buformin
N-Methyl *p*-tyramine	Santa-Cruz Biotechnology	4.42	152.100	121.200 (103.100)	51	17 (31)	8 (6)	Buformin
Norepinephrine	Santa-Cruz Biotechnology	2.99	170.200	107.000 (152.000)	36	28 (10)	20 (10)	Buformin
Normetanephrine	Sigma-Aldrich	3.32	184.100	166.000 (134.000)	36	9 (25)	10 (8)	Buformin
Norphenylephrine	Sigma-Aldrich	3.31	154.180	91.100 (136.000)	39	29 (11)	16 (8)	Buformin
Octopamine	Sigma-Aldrich	3.02	154.000	91.000 (136.000)	36	29 (11)	16 (8)	Buformin
Phenylephrine	Toronto Research Chemicals	3.93	168.210	91.000 (77.000)	41	30 (56)	11 (4)	Buformin
Salsolinol	Sigma-Aldrich	3.96	180.080	117.100 (145.200)	81	31 (25)	8 (10)	Buformin
Serotonin	Sigma-Aldrich	5.37	177.100	160.100 (132.000)	53	17 (30)	10 (10)	Serotonin-d4
Synephrine	Sigma-Aldrich	3.20	167.691	149.900 (135.000)	36	35 (35)	12 (8)	Buformin
m-Tyramine	Toronto Research Chemicals	4.30	138.100	121.100 (64.000)	31	15 (37)	8 (12)	Buformin
p-Tyramine	Sigma-Aldrich	4.16	138.100	121.100 (77.000)	43	14 (36)	12 (15)	Buformin
Buformin	Wako Chemicals	4.00	158.000	60.000 (47.000)	40	35 (66)	10 (8)	-
Serotonin-d4	Santa-Cruz Biotechnology	5.37	181.100	164.100 (136.200)	46	15 (32)	10 (8)	-

**8% organic additive** (91.9% H2O, 0.1 % formic acid, 6.9% acetonitrile, 1.1% methanol)								
4-Hydroxybenzylamine	Sigma-Aldrich	2.96	124.100	77.000 (107.000)	29	35 (9)	14 (6)	Aciclovir
Frovatriptan	Sigma-Aldrich	5.11	244.300	213.000 (170.100)	56	19 (34)	14 (10)	Ranitidin-d6
N-Methyl serotonin	Toronto Research Chemicals	4.06	191.101	160.000 (148.000)	60	17 (17)	10 (10)	Aciclovir
Phenylethylamine	Sigma-Aldrich	3.66	122.130	77.000 (79.000)	44	41 (31)	6 (16)	Metaproterenol
Pirbuterol	Sigma-Aldrich	3.70	241.300	167.200 (149.100)	65	24 (30)	15 (15)	Aciclovir
Salbutamol	Sigma-Aldrich	3.88	240.200	148.200 (222.200)	60	24 (24)	15 (15)	Aciclovir
Sotalol	Santa-Cruz Biotechnology	4.11	273.370	255.100 (133.100)	61	17 (37)	16 (8)	Aciclovir
Sumatriptan	Sigma-Aldrich	6.52	296.200	58.200 (251.200)	50	30 (24)	12 (12)	Aciclovir
Terbutaline	Sigma-Aldrich	3.98	226.200	152.100 (107.000)	60	23 (40)	10 (10)	Aciclovir
Tryptamine	Sigma-Aldrich	6.11	161.060	144.000 (117.000)	41	13 (31)	10 (8)	Ranitidin-d6
Aciclovir	Sigma-Aldrich	3.78	225.980	151.900 (134.930)	46	17 (40)	10 (8)	-
Metaproterenol	Sigma-Aldrich	3.66	212.130	152.000 (107.000)	56	23 (39)	10 (8)	-
Ranitidine-d6	Toronto Research Chemicals	4.09	321.200	176.000 (130.100)	65	25 (35)	15 (15)	-
**20% organic additive** (79.9% H2O, 0.1 % formic acid, 17.2% acetonitrile, 2.8% methanol)								
5-Methoxy tryptamine	Sigma-Aldrich	3.50	191.035	174.100 (159.100)	46	13 (31)	10 (10)	Tulobuterol
Clenbuterol	Sigma-Aldrich	4.84	277.110	258.900 (203.000)	66	15 (23)	18 (14)	Tulobuterol
Dobutamine	Sigma-Aldrich	4.22	302.202	137.000 (106.900)	66	30 (37)	10 (6)	Tulobuterol
Eletriptan	Sigma-Aldrich	12.70	383.300	144.000 (84.000)	85	40 (40)	10 (16)	Talinolol
Fenoterol	Sigma-Aldrich	3.50	304.100	107.100 (135.200)	70	44 (24)	12 (12)	Tulobuterol
Formoterol	Sigma-Aldrich	4.97	345.200	149.100 (121.100)	70	28 (42)	15 (15)	Tulobuterol
mIBG	Sigma-Aldrich	5.69	276.090	217.000 (90.000)	76	29 (54)	14 (16)	Tulobuterol
MPP+	Sigma-Aldrich	3.51	170.016	128.100 (102.200)	100	42 (63)	8 (6)	Tulobuterol
N-Methyl phenylethylamine	LGC Standards	3.48	136.060	105.100 (103.000)	56	19 (33)	20 (20)	Tulobuterol
N-Methyl tryptamine	Toronto Research Chemicals	3.58	175.105	144.000 (132.000)	61	17 (15)	8 (8)	Tulobuterol
Ractopamine	Sigma-Aldrich	4.21	302.400	107.000 (91.000)	56	43 (58)	6 (16)	Tulobuterol
Rizatriptan	Sigma-Adlrich	3.49	270.000	201.000 (158.000)	55	17 (28)	11 (11)	Tulobuterol
Tranylcypromin	Sigma-Aldrich	3.50	134.048	117.000 (115.000)	41	13 (27)	8 (8)	Tulobuterol
Zolmitriptan	Sigma-Aldrich	3.49	288.000	243.000 (182.000)	75	24 (35)	12 (12)	Tulobuterol
Talinolol	Santa-Cruz Biotechnology	11.20	364.245	308.300 (100.100)	85	27 (33)	10 (8)	-
Tulobuterol	Santa-Cruz Biotechnology	4.69	228.125	153.900 (119.100)	60	23 (41)	10 (8)	-
